# Modulators of gene amplification alter evolution of antibiotic resistance in *Staphylococcus aureus*

**DOI:** 10.1371/journal.pgen.1012011

**Published:** 2025-12-31

**Authors:** Kalinga Pavan T. Silva, Anthony M. Martini, Anupama Khare

**Affiliations:** Laboratory of Molecular Biology, National Cancer Institute, National Institutes of Health, Bethesda, Maryland, United States of America; Michigan State University, UNITED STATES OF AMERICA

## Abstract

Gene amplification is thought to be common in bacterial populations, providing a rapid and reversible mode of adaptation to diverse stresses, including the acquisition of antibiotic resistance. We previously showed that the opportunistic pathogen *Staphylococcus aureus* evolves resistance to the dual-targeting fluoroquinolone delafloxacin (DLX) that inhibits both the DNA gyrase and DNA topoisomerase IV via amplification of an efflux pump encoding gene *sdrM*. However, the pathways that control gene amplification, and consequently adaptive trajectories, remain understudied, especially in gram-positive bacteria like *S. aureus*. Here, we show that specific DNA repair and chromosomal separation proteins alter the frequency of gene amplification and selection of amplified regions in *S. aureus*. Through a screen of 40 mutants deficient in various DNA processes, we determined that while *sdrM* amplification was still the almost universal path to DLX resistance, other mutations that increased *sdrM* expression reduced the selection frequency of *sdrM* amplification, demonstrating the critical role of *sdrM* in DLX resistance. We found that similar to other bacteria, both *sdrM* amplification and loss of amplified gene copies required a functional RecA recombinase, but multiple other mutants in pathways required for amplification in other species still exhibited frequent *sdrM* amplification, suggesting that *S. aureus* may have alternate routes of gene amplification. Finally, loss of function mutants of the tyrosine recombinase XerC, that is known to play a role in chromosomal separation, were deficient for *sdrM* amplification, indicating that XerC is a novel modulator of gene amplification, or the maintenance or selection of amplified gene copies. Thus, our work sheds light on genetic factors that alter gene amplification-mediated evolutionary trajectories to antibiotic resistance in *S. aureus* and can potentially unlock mechanisms by which such evolution of resistance can be inhibited.

## Author summary

Bacteria can become resistant to antibiotics by making tandem copies of segments of their DNA that contain resistance genes in a process called gene amplification. We had previously shown that the pathogen *Staphylococcus aureus* becomes resistant to the clinically relevant antibiotic delafloxacin (DLX) by gene amplification of *sdrM*, that codes for an efflux pump. Additional copies of *sdrM* increase the amount of DLX removed from the cell, resulting in *S. aureus* cells resistant to DLX. However, the mechanisms of gene amplification in *S. aureus* are not known. In this study, we used experimental evolution of a targeted panel of mutants to determine that two recombinase enzymes that mediate exchange of DNA, RecA and XerC, are critical for gene amplification in *S. aureus*. While RecA is required for gene amplification in other bacteria, XerC had not been previously implicated in gene amplification. We further found that mutations that increase expression of *sdrM* provide another path to DLX resistance, highlighting the importance of *sdrM* expression for DLX resistance in *S. aureus*. Our work sheds light on the vital determinants of gene amplification and can aid in the development of therapeutics against the emergence of antibiotic resistance in *S. aureus*.

## Introduction

Many antibiotics used to treat bacterial infections are growing increasingly ineffective as clinical and environmental bacteria acquire resistance via horizontal gene transfer or *de novo* mutation-mediated evolution [1,2]. Such antimicrobial resistance is a major threat to global public health [3,4]. *Staphylococcus aureus*, especially methicillin-resistant *S. aureus* (MRSA), commonly acquires resistance to a broad range of antibiotics, and is a major cause of antibiotic resistant infections world-wide [5,6].

While many instances of mutation-driven evolution of antibiotic resistance involve sequence alterations, gene amplification can also lead to antibiotic resistance via a variety of mechanisms [7,8]. Gene amplification has been previously implicated in resistance to multiple antibiotics in *S. aureus*. Duplication of the gene encoding the efflux pump *norA* led to ciprofloxacin resistance [9], amplification of the SCC*mec* cassette caused oxacillin resistance [10], and amplification of other genomic regions resulted in resistance against macrolides [11] and intermediate resistance against vancomycin [12].

Delafloxacin (DLX) is a dual-targeting antibiotic capable of inhibiting the activities of the DNA gyrase and DNA topoisomerase IV in multiple bacterial species, including *S. aureus* [8,13]. Such antibiotics that target two or more components in the cell should theoretically lead to a lower frequency of resistance evolution, as cells would need mutations in multiple targets for resistance [14,15]. In an earlier study, we identified two distinct mechanisms leading to high DLX resistance in *S. aureus*. The more rapid and prevalent evolutionary trajectory was via gene amplification of the gene encoding an efflux pump, SdrM, that increased DLX efflux from the cell, while the second adaptive path, that we observed in the absence of functional *sdrM*, was through mutations in both the canonical DLX targets, DNA gyrase and DNA topoisomerase IV [16].

Previous work has shown a significant reduction in duplication and amplification frequency in mutants deficient for the DNA recombinase RecA in multiple bacterial species upon exposure to selective pressures [17,18]. Apart from RecA, other proteins involved in DNA double-strand break (DSB) repair and some involved in other DNA metabolic processes are critical for gene amplification in *E. coli* [19]. However, factors that may alter the frequency of gene amplification, or maintenance and selection of amplified genomic loci, and thus evolutionary trajectories of antibiotic resistance, have not been characterized in gram-positive bacteria including *S. aureus*.

Here, we investigate the genes and pathways involved in *sdrM* gene amplification in *S. aureus*. We find that RecA is required for gene amplification in *S. aureus*. In the absence of functional RecA, *S. aureus* requires two steps to evolve DLX resistance, where the initial mutations partially compensate for the DNA damage sensitivity in the *recA* mutant, and are followed by mutations in *sdrM* or the canonical DLX targets. RecA also regulates the maintenance of amplified *sdrM* copies, as these are more stable in the absence of functional RecA. Further, through a targeted screen of mutants in various DNA repair and metabolism pathways, we observe that *sdrM* amplification remained the pervasive mechanism of DLX resistance, even in the absence of proteins involved in the DSB repair pathway that is required for gene amplification in *E. coli*. However, the evolution of point mutations that increase expression of *sdrM* selects against *sdrM* amplification, indicating that albeit less frequent, specific sequence alterations that achieve increased *sdrM* expression can lead to a lower selective advantage of *sdrM* amplification, and highlighting the importance of SdrM function in conferring DLX resistance. Finally, we find that the widely conserved tyrosine recombinase XerC, involved in chromosomal separation, is a novel effector of *sdrM* amplification.

## Results

### Resistance evolution via gene amplification requires the recombinase RecA

Gene amplification is thought to be a two-step process where an initial duplication event is mediated by either homologous or non-homologous recombination, and is followed by higher order amplification via RecA*-*mediated homologous recombination [8]. Thus, while the initial duplication may be RecA-dependent or independent, RecA is thought to be essential for the subsequent gene amplification [20]. To test whether this is also valid for gene amplification in *S. aureus* and determine how the absence of RecA alters evolutionary trajectories of DLX resistance, we evolved the *S. aureus* JE2 *recA*::Tn mutant from the Nebraska Transposon Mutant Library (NTML) [21] for DLX resistance. The *recA*::Tn mutant is ~ 56-fold more sensitive to DLX compared to the WT (S1 Fig). Fluoroquinolones cause DNA DSBs [22] and DLX is a dual-targeting fluoroquinolone that targets both the DNA gyrase and topoisomerase IV enzymes. Through a TUNEL assay, which allows for the detection for 3’-OH ends of fragmented DNA, we confirmed that DLX exposure also leads to DNA damage (S2 Fig). This DNA damage likely underlies the significant DLX hypersensitivity of the DNA repair defective *recA*::Tn mutant. We passaged three independent populations of *recA*::Tn in increasing concentrations of DLX, starting from an initial DLX concentration ~ 0.5x the MIC of the *recA*::Tn mutant (the MIC is ~ 0.006 µg/mL) and increasing the DLX concentration during subsequent passaging until the concentration was at least 8 µg/mL (see Methods for details). We performed whole genome sequencing (WGS) on populations from select intermediate passages as well as the terminal passage.

**Fig 1:**
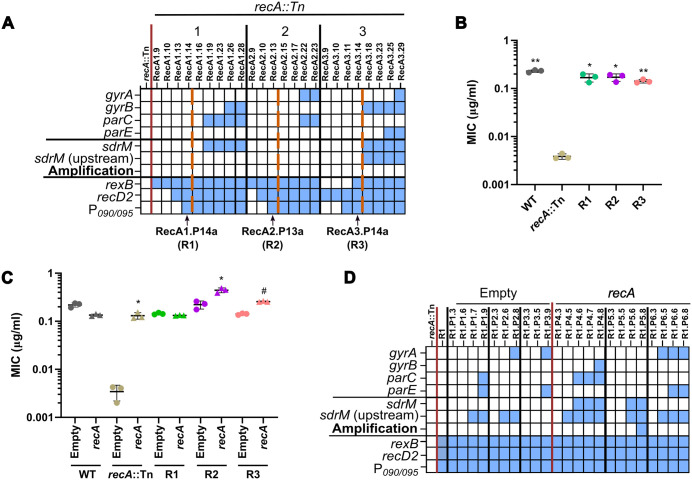
RecA is required for *sdrM* gene amplification. (A) Common mutations and *sdrM* amplification seen in three independently evolved populations of the *recA*::Tn mutant. Within each population, earlier to later passages are shown from left to right. Blue squares show the presence of a mutation in the loci listed on the y-axis, or *sdrM* amplification, as indicated. Dashed red lines indicate when the populations reached a resistance level similar to the WT. The populations from which the ‘intermediate isolates’ were selected are shown by black arrows. (B) DLX MICs of WT, the *recA*::Tn mutant, and the three intermediate isolates. (C) DLX MICs of WT, the *recA*::Tn mutant, and the three intermediate isolates, each carrying either an empty pKK30 plasmid, or one with the *recA* gene. (D) Presence of mutations (as shown by the blue squares) in the DNA gyrase and topoisomerase IV targets, *sdrM*, and DNA repair associated loci, as well as *sdrM* amplification, in three independently evolved populations each of the R1 intermediate isolate with either the pKK30 empty vector or one carrying *recA*. For each population, passages are shown chronologically from left to right. (B, C) Data shown are the mean ± standard deviation of three independent biological replicates. Significance is shown for comparison to (B) the *recA*::Tn mutant and (C) the respective empty vector strain as tested by Brown-Forsythe and Welch ANOVA tests followed by the Dunnett’s T3 multiple comparison’s test (* *p* < 0.05, ** *p* < 0.01, # *p <* 0.0001).

None of the three *recA*::Tn populations showed *sdrM* gene amplification in any of the sequenced passages (Fig 1A). Instead, mutations in the canonical targets DNA gyrase (*gyrA*, *gyrB*), and topoisomerase IV (*parC*, *parE*), and in and upstream of *sdrM* were prevalent in the later passages (Fig 1A and S1 Table). Interestingly, these mutations were selected during the evolution only once the evolving populations had reached a DLX resistance level similar to that of the wild type (WT), while mutations in genes associated with DNA repair were observed in the early passages. Individual isolates from each population (R1, R2, and R3 from populations 1, 2, and 3 respectively) from a passage prior to the emergence of the canonical mutations had DLX resistance similar to that of the WT (Fig 1B). Further, complementing RecA back into these isolates resulted in only an up to ~2-fold increase in DLX resistance, compared to the ~ 38-fold increase seen in the *recA*::Tn mutant (Fig 1C), suggesting that these isolates may have recouped the intrinsic DLX resistance lost due to the nonfunctional RecA via mutations in genes different from the canonical targets or *sdrM*.

To further clarify if RecA is required for gene amplification, we evolved three populations of the intermediate isolate R1 complemented with RecA, in parallel to three populations of R1 with an empty vector control. Given that the DLX MIC for R1 was similar to the WT, and it did not have any of the canonical mutations, we reasoned that if RecA was necessary for amplification, re-introducing a functional RecA and selecting for DLX resistance should lead to *sdrM* amplification. One of the three RecA-complemented populations showed *sdrM* gene amplification, while none of the empty vector control populations did (Fig 1D and S2 Table), further validating that RecA is required for *sdrM* amplification upon selection for DLX resistance.

### Absence of functional RecA results in a two-step evolution of DLX resistance

**Fig 2:**
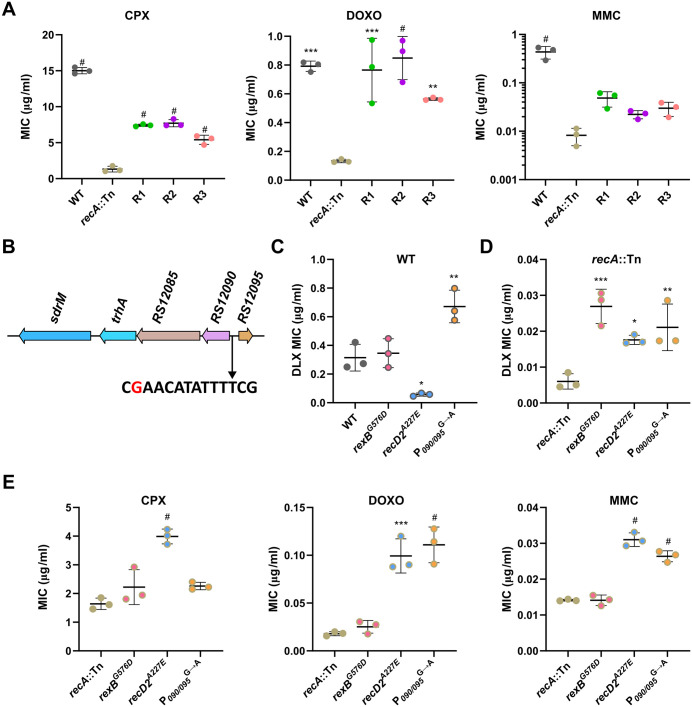
Mutations in *recA*::Tn intermediate isolates reduce DLX and DNA damage sensitivity. (A) MICs for ciprofloxacin (CPX), doxorubicin (DOXO), and mitomycin C (MMC) of WT, the *recA*::Tn mutant, and the three intermediate isolates. (B) The position of a G to A mutation (shown in red) in the putative LexA-binding site (shown in black) located between the genes *B7H15_RS12090* and *B7H15_RS12095* (P_*090/095*_). (C) DLX MICs for WT and the indicated allele-replacement strains in the WT background. (D) DLX MICs for the *recA*::Tn mutant and the indicated allele-replacement strains in the *recA*::Tn background. (E) MICs for ciprofloxacin (CPX), doxorubicin (DOXO), and mitomycin C (MMC) of the *recA*::Tn mutant and the indicated allele-replacement strains in the *recA*::Tn background. (A, C-E) Data shown are the mean ± standard deviation of three independent biological replicates. Significance is shown for comparison to (A) the *recA*::Tn mutant, (C-E) the respective parental strain as tested by a one-way ANOVA with Dunnett’s test for multiple comparisons (* *p* < 0.05, ** *p* < 0.01, *** *p* < 0.001, # *p <* 0.0001).

RecA plays two important roles in DNA repair, first by activating the SOS response induced by DNA damaging agents, and second by acting as a critical effector of homologous recombination-mediated DSB repair [23,24]. Given that DLX also leads to DNA damage (S2 Fig), we hypothesized that the mutations in the DNA repair associated genes seen in the intermediate isolates may be reducing the DNA damage susceptibility of the *recA*::Tn mutant. Therefore, we tested the susceptibility of the intermediate isolates against multiple DNA damaging agents. We found that the intermediate isolates not only showed lower DLX susceptibility compared to the *recA*::Tn mutant, but also had reduced susceptibility against three other DNA damaging agents, a different fluoroquinolone ciprofloxacin (CPX) [25,26], the anti-cancer drug doxorubicin (DOXO) [27] and the chemotherapeutic mitomycin C (MMC) [28], compared to the hyper-sensitive *recA*::Tn mutant (Fig 2A).

Given that the intermediate isolates showed reduced susceptibility to DLX and DNA damaging agents, but do not have canonical DLX resistance mutations, we investigated common mutations seen in these isolates, which were also seen in the early passages of the *recA*::Tn evolution (Fig 1A). All three isolates had coding sequence mutations in the genes encoding the helicase RexB, and a RecD2-like helicase, as well as an intergenic mutation upstream of the genes *B7H15_RS12090* and *B7H15_RS12095* (P_*090/095*_) (Table 1). RexB is involved in the processing of double stranded breaks (DSBs) during DSB repair, similar to the activity of RecBCD in most gram-negative bacteria [23]. The two mutations observed in RexB, E48K and G576D, are both in residues that are conserved across species (S3A Fig), and the E48 residue in the *Bacillus subtilis* homolog AddB has been implicated in binding to DNA [29], indicating that mutations in these residues likely alter RexB function. While the role of the RecD2 helicase has not been characterized in *S. aureus*, RecD2 is present in other bacteria that lack RecBC and is thought to be involved in varied DNA repair processes [30–32]. In the intermediate isolates, we observed two different mutations, G360A and A227E, but the evolving populations showed numerous other coding sequence mutations, including A346D, T367P, T368T, T420M, D449G, Q480H, and N629K, which are all in conserved residues (S1 Table and S3B Fig). Given the diversity of residues, the effect of these mutations on DNA repair is unclear.

**Table 1:** Common mutations in the three *recA*::Tn evolved intermediate isolates.

Strain	*rexB*	*recD2*	P_*090/095*_ (at 2,306,688)
**R1**	E48K	G360A	G → A
**R2**	G576D	A227E	G → A
**R3**	G576D	A227E	G → A

The third mutation we observed in all three intermediate isolates was an identical G to A substitution in the intergenic region between the adjacent divergently encoded genes, *B7H15_RS12090* and *B7H15_RS12095* (P_*090/095*_). *B7H15_RS12090* is in the SOS regulon of *S. aureus* along with the two immediately downstream genes located in the same operon (*B7H15_RS12085* and *trhA*) [23]. The intergenic mutation (P_*090/095*_^G→A^) lies within the predicted LexA binding site in this region [25] (Fig 2B) and may thus alter the regulation of these three genes.

To test the role of each mutation in conferring reduced DLX susceptibility in the *recA*::Tn background, we transferred *rexB*^*G576D*^, and *recD2*^*A227E*^, which were both found in two independently evolved intermediate isolates each, as well as the P_*090/095*_^G→A^ mutation, individually to both the WT and *recA*::Tn backgrounds. We observed that while only the P_*090/095*_^G→A^ mutation conferred a ~ 2-fold increase in DLX resistance in the WT background, (Fig 2C), all three mutations individually led to reduced DLX susceptibility in the DLX-hypersensitive *recA*::Tn mutant background (Fig 2D), accounting for their selection during the evolution.

We also tested the role of the individual mutations in alleviating DNA damage sensitivity. We found that in the *recA*::Tn background, while the *recD2*^*A227E*^ allele-replacement strain was less susceptible to all three DNA damaging agents, and the P_*090/095*_^G→A^ mutation led to reduced susceptibility against DOXO and MMC, *rexB*^*G576D*^ did not significantly alter resistance against any of the agents, indicating a specific effect on DLX susceptibility in the absence of functional RecA (Fig 2E). Thus, in the absence of *sdrM* amplification, the *recA*::Tn mutant evolves DLX resistance via a two-step process: initial mutations associated with DNA repair genes that partially compensate for its DNA damage sensitivity, followed by point mutations in the DNA gyrase, DNA topoisomerase IV, and *sdrM*.

### RecA is required for loss of amplified copies of *sdrM*

**Fig 3:**
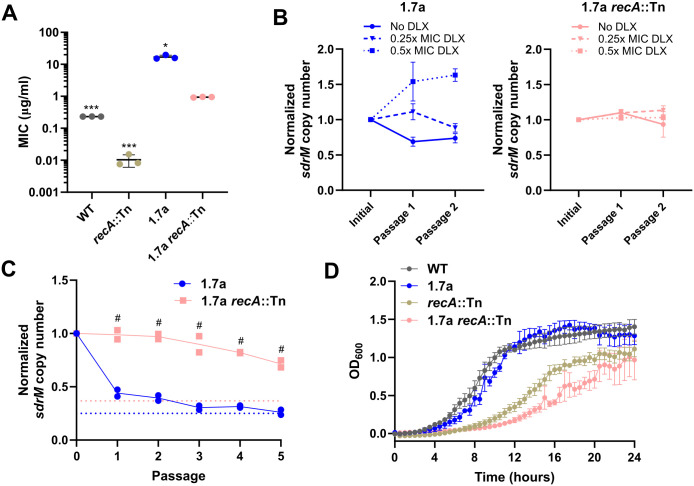
Amplified segments are stabilized in the absence of RecA and have a fitness cost. (A) DLX MICs for WT, and 1.7a, as well as *recA*::Tn mutants in both backgrounds. Data shown are the mean ± standard deviation of three independent biological replicates. (B) Normalized *sdrM* copy number (normalized to the copy number in the initial culture) for 1.7a (left) and 1.7a *recA*::Tn (right) upon passaging in either no DLX, or in two different DLX concentrations, corresponding to ~0.25x and ~0.5x the respective MICs. The data for 1.7a are from our previous study [16] licensed under the Creative Commons Attribution 4.0 International License and are shown here as *sdrM* copy number normalized to the initial value (instead of absolute *sdrM* copy number as in the original figure). Data shown are the mean ± standard deviation of two independent passaging experiments. (C) Normalized *sdrM* copy number (normalized to the copy number in the respective initial culture) for 1.7a and 1.7a *recA*::Tn upon extended passaging without DLX. The dotted lines denote an absolute copy number of 1 for the respective samples. Data from two independent passaging experiments are shown. (D) Growth curves shown as OD_600_ measurements of WT, and 1.7a, as well as *recA*::Tn mutants in both backgrounds. Data shown are the mean ± standard error of the mean of three independent biological replicates. Significance is shown for comparison to (A) the 1.7a *recA*::Tn strain, and (C) the respective 1.7a passage as tested by (A) Brown-Forsythe and Welch ANOVA tests followed by the Dunnett’s T3 multiple comparison’s test or (C) a two-way ANOVA with Sidak’s multiple comparisons test (* *p* < 0.05, *** *p* < 0.001, # *p <* 0.0001).

To test whether RecA plays a role in the instability of the amplified *sdrM* locus, we introduced the *recA*::Tn mutation to an evolved strain from our previous study, 1.7a, which had *sdrM* amplification leading to a ~ 140-fold increase in DLX resistance compared to the WT [16]. The 1.7a *recA*::Tn strain had a ~ 18-fold reduction in DLX resistance compared to the parental 1.7a strain, and a ~ 94-fold higher DLX resistance compared to the *recA*::Tn strain (Fig 3A). We passaged this strain in different concentrations of DLX, and found that unlike what we had previously seen in the 1.7a mutant [16] (shown here in the left panel of Fig 3B), the copy number of *sdrM* did not show significant variability in the 1.7a *recA*::Tn mutant (Fig 3B), suggesting that RecA likely plays a role in the expansion of the *sdrM* amplification. Further, we passaged 1.7a and 1.7a *recA*::Tn in DLX-free media for five passages and found that the normalized *sdrM* copy number compared to the initial passage remained significantly higher in the 1.7a *recA*::Tn strain compared to in 1.7a, where the copy number reached ~1 indicating an almost complete loss of the amplified copies of the locus (Fig 3C).These data validate that RecA is critical for the loss of amplified copies of *sdrM*. Given the stability of the amplified *sdrM* locus in the 1.7a *recA*::Tn strain, we tested its growth in DLX-free media to determine the effect of amplified *sdrM* copies on fitness. We found that while 1.7a showed similar growth compared to the WT, and the *recA*::Tn mutant grew slower than those strains, the 1.7a *recA*::Tn mutant showed substantially reduced growth even compared to the *recA*::Tn mutant, indicating a significant fitness cost of the amplified locus, at least in the absence of functional RecA (Fig 3D and S4 Fig).

### Mutant library screen shows that evolution of DLX resistance leads to prevalent *sdrM* amplification with diverse junctions

The absence of functional SdrM alters DLX resistance trajectories by requiring dual target mutations in the DNA gyrase and topoisomerase IV [16], while the absence of RecA leads to a two-step evolution of DLX resistance (Figs 1 and 2). To identify other genes that modulate the formation or selection of an amplified *sdrM* locus, and thereby the evolutionary trajectory of DLX resistance, we considered genes involved in DNA repair and recombination, the SOS regulon, and chromosomal separation, and those located in the genomic neighborhood of *sdrM*. We identified 39 genes of interest that had available loss of function transposon mutants in the NTML [21] (S3 Table). We also constructed two mutants that had *lexA* alleles (*lexA*^*S130A*^ and *lexA*^*G94E*^) previously reported to encode uninducible versions of *lexA*, thereby inhibiting the SOS response [25,33,34]. We determined the DLX MICs of the corresponding mutants (S1 Fig) and for a majority of mutants, as well as a WT control, we evolved three independent populations in increasing DLX concentrations, starting from ~0.5x the respective MIC. For some mutants with very low MICs (*xerC*::Tn and *recG*::Tn), we evolved one population until its MIC was similar to the WT and then split that intermediate population into three independent populations for further evolution. We initially evolved the populations up to a DLX concentration of 2 µg/mL and tested for the presence of amplified *sdrM* using WGS. For the populations without *sdrM* gene amplification, the evolution was continued until a DLX concentration between 8–16 µg/mL.

**Fig 4:**
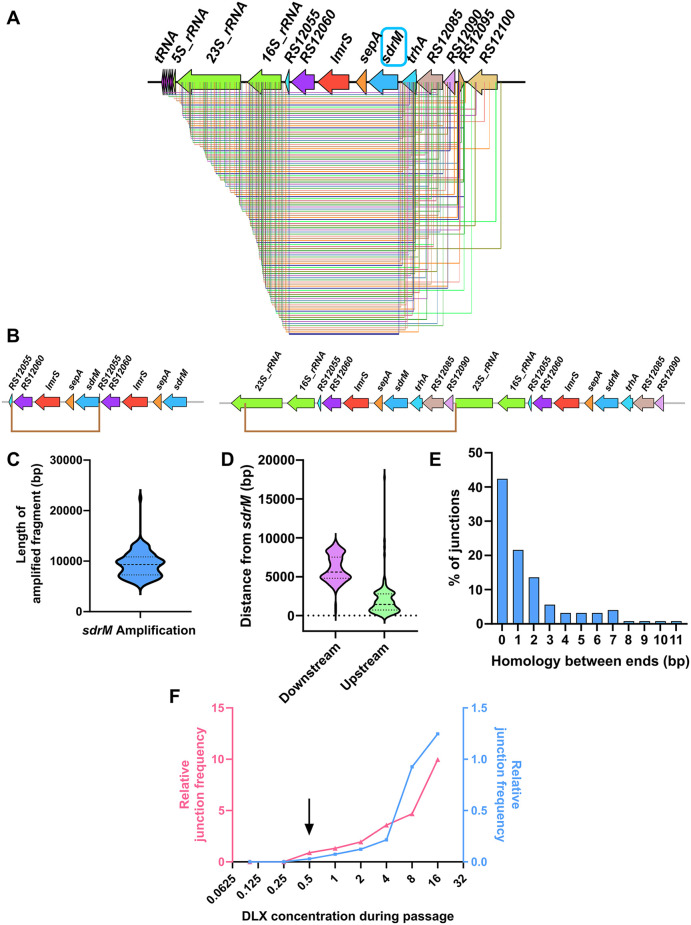
Amplified fragments containing *sdrM* are highly prevalent, diverse, and non-homologous, and originate in an rRNA-tRNA locus. (A) All distinct amplified fragments containing *sdrM* seen in our evolved populations. The terminus downstream of *sdrM* in almost all the fragments is in the tRNA-rRNA locus. (B) Two examples of *sdrM* amplification each showing two copies of the amplified fragment (bordered by the brown lines). The one on the left is among the shortest amplified fragments, whereas the one on the right is significantly longer. (C) Length distribution of the amplified fragments containing *sdrM*. (D) Distribution of the distances of the downstream and upstream ends of the amplified fragments from the coding sequence of *sdrM*. The downstream ends are significantly further away as they are concentrated in the rRNA-tRNA locus, while the upstream ends start immediately upstream of *sdrM*. (E) Distribution of the length of homology between the two ends of the amplified fragments indicates a lack of significant homology. (F) Relative frequency of novel junctions representing a duplication (compared to *rpoC* levels), as measured by qPCR, in genomic DNA from successive passages of two independently evolved WT populations. For both populations, the junctions were detected in the passage containing 0.5 µg/mL DLX (shown by the black arrow).

Given the large numbers of populations evolved, we first analyzed the general trends for DLX resistance in *S. aureus*. Of the 126 independent populations evolved, 108 showed *sdrM* amplification, reinforcing the finding that *sdrM* amplification comprises a major evolutionary path to DLX resistance. 121 distinct genomic segments around *sdrM* were amplified, each found in only one evolved population, with the exception of two amplified segments that were found in two different independently evolved populations each (S4 Table). The amplified segments were diverse both in size and exact genomic coordinates of their ends (Fig 4A-4C). While most of the upstream ends of the fragments were located in a ~ 4.7kb kb region starting ~50 bp upstream of *sdrM*, all the downstream ends were located in a ~ 6 kb region starting ~3.2 kb away from *sdrM* (Fig 4D). These downstream ends were located almost exclusively in a tRNA-rRNA cluster, located four genes downstream of *sdrM*, resulting in these four genes also being present in most amplified segments. Our previous work had showed that of all the genes present in the amplified segments, DLX resistance depended solely on the overexpression of *sdrM* [16]. The prevalence of amplicon ends in the tRNA-rRNA cluster, skipping the intermediate genes, thus indicates that the tRNA-rRNA cluster may be a hot-spot for the initiation of amplification. Further, a synteny analysis of 28 genomes from different *Staphylococcus* species and 2 genomes from closely related *Mammaliicoccus* species (S5 Table) revealed that in all genomes, *sdrM* homologs were located between ~2–5 kb away from rRNA loci (S5 Fig), indicating that this proximity is conserved. Finally, the ends of the amplified segments had limited homology (Fig 4E), indicating a role of non-homologous recombination in the formation of these junctions, as observed in the WT in our previous study [16].

Given the numerous distinct amplified segments we found, we wanted to determine whether these could be detected in the unselected parental strains. We first measured the detection limit for two novel junctions that were selected in one WT population each to be 1 in ~10^4^-10^5^ cells (S6A Fig). Further, we found that in these two WT populations, the junctions were first detected in populations being passaged at 0.5 µg/mL DLX, which is ~ 2x the WT MIC (Fig 4F), but not in the previous passages. Thus, if these junctions, which represent at least a duplication, were present in the unselected parental strain, they must exist at a frequency lower than 1 in 10^4^-10^5^ cells. Similarly, we found that the detection limit of two novel junctions that were found in one population each of two transposon mutants (*spoIIIE*::Tn and *recQ2*::Tn) was also 1 in ~10^4^-10^5^ cells (S6B, C Fig). These junctions were also not detected in the respective parental strains, indicating a comparably low frequency of 1 in 10^4^-10^5^ cells, if they existed in the preselected parental strain. To identify any other junctions in this region in the unselected WT strain, we used an inverse PCR-like strategy, in conjunction with TOPO TA cloning (S7A Fig). We identified three different junctions from three independently grown WT cultures (S7B Fig), neither of which were present in any of our evolved populations. However, we could not detect them using junction-specific primers, indicating that if they existed in the WT strain, their frequency in the population was less than 1 in ~10^4^-10^5^.

### Mutations increasing *sdrM* expression are associated with a lack of *sdrM* amplification

We next examined the mutations observed during the evolution, focusing on common mutations seen in more than one mutant background (S6 Table). Apart from amplification of *sdrM*, evolved populations from almost all mutants also showed increased coverage of a ~ 43 kb genomic region that contains multiple predicted phage proteins, as well as two toxin-antitoxin system toxins, and one anti-phage defense protein (S8A Fig). Using PHASTER, an online tool that identifies and annotates prophages [35,36], we determined that this region encodes an intact prophage (S8B Fig), suggesting that exposure to increasing DLX concentrations led to induction of this prophage.

**Fig 5:**
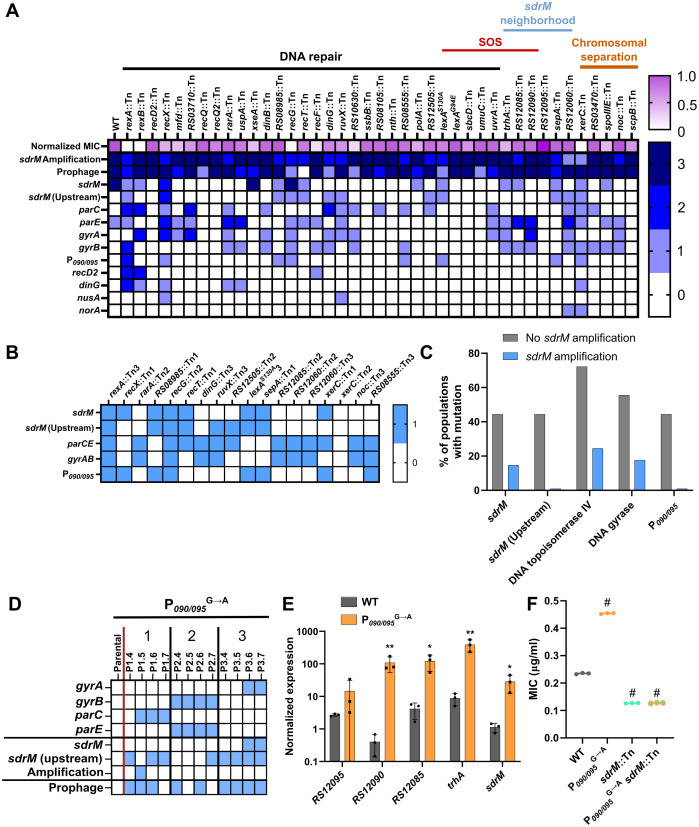
Amplification of *sdrM* is prevalent upon selection for DLX resistance but negatively associated with mutations increasing *sdrM* expression. (A) Relative MICs (normalized to the WT) and the number of independently evolved populations that showed the mutations in the indicated loci, as well as *sdrM* amplification and increased prophage coverage, for WT and the screened mutants (a complete list of mutants is in S3 Table). (B) Common mutations (indicated by the blue squares) seen in the independently evolved populations that did not contain amplified *sdrM*. (C) Percentage of populations with or without *sdrM* amplification that had mutations in the indicated loci. (D) Common mutations seen in three independently evolved populations of an allele-replacement strain carrying the P_*090/095*_^G→A^ mutation. Presence of mutations in the corresponding loci or *sdrM* amplification is indicated by a blue square. For each population, passages are shown from left to right in chronological order. (E) Normalized expression (compared to *rpoC*) of the indicated genes in the WT and the allele replacement strain carrying the P_*090/095*_^G→A^ mutation. (F) DLX MICs for WT, the P_*090/095*_^G→A^ mutant, and the *sdrM*::Tn mutant in both backgrounds. (E, F) Data shown are the mean ± standard deviation of three independent biological replicates. Significance is shown for comparison to (E) the respective WT sample as tested by ratio paired *t-*tests, and (F) the WT as tested by a one-way ANOVA with Dunnett’s test for multiple comparisons (* *p* < 0.05, ** *p* < 0.01, # *p <* 0.0001).

Similar to our previous study [16], many evolved populations contained mutations in the canonical DNA gyrase and topoisomerase IV targets and in *sdrM* (Fig 5A). Apart from the previously identified coding sequence mutations in *sdrM* (A268S and Y363H) that increase DLX efflux and resistance [16], we also observed additional *sdrM* mutations that emerged including T180I, Y363F, Y363N, K390I, K390N, K390T, and an in-frame 3 bp deletion resulting in removal of the threonine at position 8 (S6 Table), which may be novel alleles of *sdrM* that confer DLX resistance. A few other common mutations were coding sequence and intergenic upstream alterations in genes associated with DNA damage and repair (*recD2*, *dinG*) [32,37]*,* bacterial transcription (*nusA)* [38] and antibiotic resistance (*norA*) [9], which may play a role in DLX resistance at least in the specific mutant backgrounds where they were selected.

Of the 126 evolved populations, 18 did not show *sdrM* amplification. In these populations, we observed mutations in the DLX targets (DNA gyrase and topoisomerase IV), coding and upstream mutations in *sdrM*, and mutations in the intergenic region upstream of the genes *B7H15_RS12090* and *B7H15_RS12095* (P_*090/095*_) (Fig 5B). For the intergenic region, most populations had the same G to A substitution (P_*090/095*_^G→A^) in the LexA-binding site seen in the *recA*::Tn populations (Fig 2B), while one population had a single base-pair deletion at the same position. While the frequency of all these mutations was higher in the populations without *sdrM* amplification (Fig 5C), mutations upstream of *sdrM*, and in P_*090/095*_ were almost exclusively seen in evolved populations without *sdrM* amplification. Additionally, in an allele-replacement strain carrying the P_*090/095*_^G→A^ mutation, only one of three populations selected for DLX resistance showed *sdrM* amplification. Further, the amplified fragments emerged transiently and disappeared, which was a rare observation across any of our previous evolutions in other strains (Fig 5D and S7 Table). Thus, the presence of the P_*090/095*_^G→A^ mutation likely reduces the selection of amplified *sdrM* fragments upon DLX exposure.

Three genes adjacent to this intergenic region are thought to be in the SOS regulon, and their expression is likely to be affected by mutations in the LexA binding site [25]. We found that the expression of these genes as well as the adjacent genes *sdrM* and *B7H15_RS12095* is significantly higher in the presence of the P_*090/095*_^G→A^ mutation, compared to the WT strain (Fig 5E). Further, while a P_*090/095*_^G→A^ mutant has higher DLX resistance (Fig 2C), when we introduced the *sdrM*::Tn mutation into that background, the DLX resistance level decreased to that of an *sdrM*::Tn mutant, indicating that the increased resistance in the P_*090/095*_^G→A^ mutant is likely solely due to *sdrM* overexpression (Fig 5F).

Additionally, in a mutant containing an *sdrM* intergenic mutation at the -164 position along with the A268S coding sequence mutation that we had previously studied [16], *sdrM* expression was significantly higher compared to a mutant containing only the A268S mutation (S9 Fig). The mutations we see in the *sdrM* intergenic region in our evolved populations are mostly clustered between positions -145 to -148 and -161 to -164 (S6 Table) and accordingly may also confer DLX resistance by increasing *sdrM* expression. Thus, mutations that increase *sdrM* expression likely reduce the selective advantage of *sdrM* amplification, providing alternate trajectories of DLX resistance evolution.

### XerC, but not the DSB repair pathway, is a major modulator of *sdrM* gene amplification

Of the 41 mutants tested, 39 showed *sdrM* amplification in at least two of the three evolved populations (Fig 5A), suggesting that the proteins encoded by the genes that are mutated in these strains may not play a major role in amplification. However, minor roles cannot be excluded given the small number of independently evolved populations for each mutant. These proteins include RexA and RexB, which together play a role in DSB repair similar to RecBCD in *E. coli*, that has been implicated in gene amplification [19]. This suggests that the mechanisms of gene amplification in *S. aureus* may be different from those in *E. coli*.

We also tested mutants in multiple DNA polymerases, helicases, and nucleases that are thought to be involved in DNA repair and recombination [23,39], and most of these mutants amplified *sdrM* in at least two of the three evolved populations. Further, the two uninducible LexA alleles did not alter the frequency of gene amplification, indicating that the SOS response does not play a significant role in gene amplification, similar to what has been reported in *E. coli* [40]. However, two mutants, *xerC*::Tn, and *B7H15_RS12060*::Tn had only one independent population each with amplified *sdrM*, indicating a potential defect in gene amplification.

XerC is a widely conserved tyrosine recombinase that is critical for the separation of chromosomes [41], and *B7H15_RS12060* encodes a putative P-Loop NTPase that is located in proximity to *sdrM*. To further test whether these genes played a role in gene amplification, we conducted a secondary screen. We evolved 12 independent populations of each mutant in a 96-deep well plate for 14 days, where each population was passaged daily in a serial dilution of DLX, and the population growing in the highest DLX concentration was propagated to the next passage (S10 Fig). As controls, we also performed a similar evolution for the WT, and for a mutant (*B7H15_RS12085*::Tn) that showed *sdrM* amplification in two out of three populations in our initial tube evolution (S6 Table).

**Fig 6:**
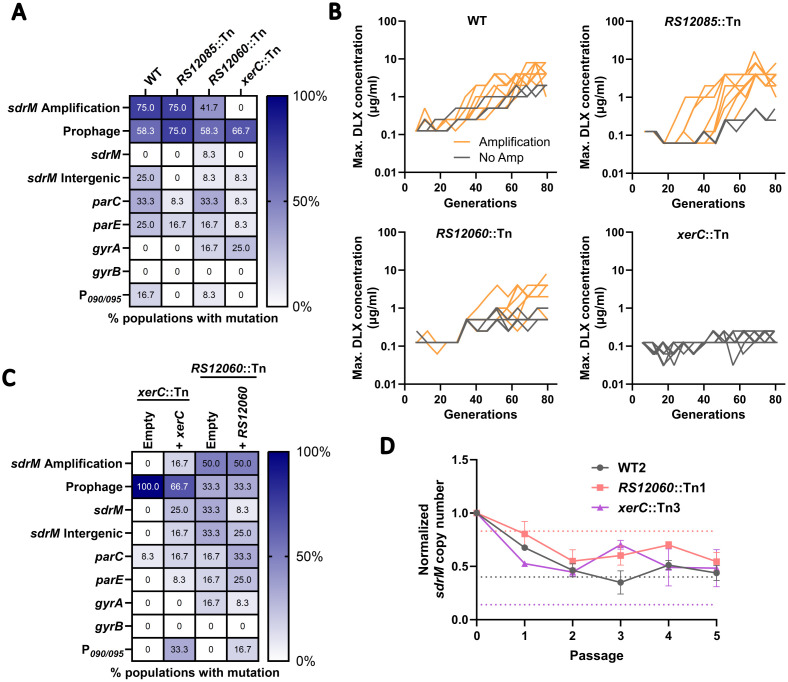
XerC is a novel determinant of amplification. (A) Percentage of 12 independently evolved populations of the WT, *RS12085*::Tn, *RS12060*::Tn, and *xerC*::Tn that showed *sdrM* amplification, putative prophage induction, or mutations in the indicated genes. (B) Maximum DLX concentration that showed growth at each passage (shown as the respective number of generations) for the 12 independently evolved populations of WT, *RS12085*::Tn, *RS12060*::Tn, and *xerC*::Tn. Orange lines represent populations with *sdrM* amplification, while grey lines represent populations without *sdrM* amplification. (C) Percentage of 12 independently evolved populations of the *xerC*::Tn and *RS12060*::Tn strains complemented with either an empty vector or one containing the respective gene, that showed *sdrM* amplification, putative prophage induction, or mutations in the indicated loci. (D) Normalized *sdrM* copy number (normalized to the copy number in the initial culture) upon extended passaging without DLX for an evolved population each of WT, *RS12060*::Tn, and *xerC*::Tn showing *sdrM* amplification. Dotted lines denote the absolute copy number for the respective samples. Data shown are the mean ± standard deviation of two independent passaging experiments.

For both the WT and the control mutant, nine out of the 12 evolved populations contained amplified *sdrM* (Fig 6A and S8 Table), indicating that mutants that amplified *sdrM* in two out of three evolved populations in the original tube evolution may not have major defects in gene amplification. For the *B7H15_RS12060*::Tn strain, only five out of twelve populations amplified *sdrM*, while none of the *xerC*::Tn mutant populations showed gene amplification (Fig 6A). Further, in this resistance evolution protocol, most evolved populations without *sdrM* amplification did not attain significant resistance (Fig 6B).

The *B7H15_RS12060*::Tn and *xerC*::Tn mutants have transposon insertions in the respective genes, but the insertions could possibly cause polar effects, especially for *xerC*, which is the first gene in an operon that contains *hslUV*, encoding a heat-shock responsive quality control protease [42], and *codY*, encoding a master regulator of metabolism and virulence [43]. To therefore test whether *xerC* and *B7H15_RS12060* were involved in amplification, we complemented the respective genes back on a plasmid and repeated the deep well evolution (S8 Table). We observed no change in the frequency of gene amplification upon complementation of *B7H15_RS12060*, but an increased frequency upon *xerC* complementation, demonstrating that *xerC* is a determinant of gene amplification (Fig 6C). Finally, we passaged an evolved population containing amplified *sdrM* from each mutant background (from the tube evolutions) in DLX-free medium and observed no significant difference in the rate of loss of amplified copies of *sdrM* between the three strains (Fig 6D). However, while the absolute copy number in the WT and *B7H15_RS12060*::Tn backgrounds reached a value of 1 or less, indicating a complete loss of amplified copies of *sdrM*, the absolute copy number remained high in the *xerC*::Tn background, indicating a possible role of XerC in the maintenance of *sdrM* amplicons (Fig 6D).

### The role of XerC in gene amplification is independent of RecA expression

**Fig 7:**
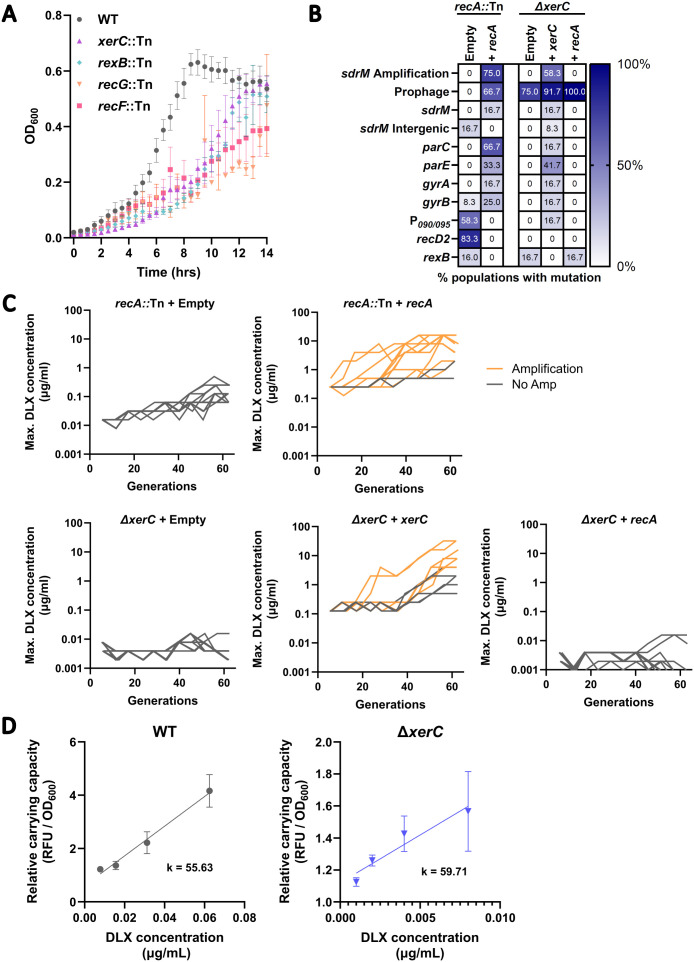
XerC promotes gene amplification in a RecA-independent manner. (A) The indicated strains were diluted 1:100 in fresh medium, and OD_600_ was measured every 30 minutes. Data shown are the mean ± SEM for three independent biological replicates. (B) Percentage of 12 independently evolved populations of the *recA*::Tn strain complemented with either an empty vector or one containing P_*recA*_::*recA*, and the Δ*xerC* mutant complemented with either an empty vector, or one containing P_*xerC*_::*xerC* or P_*tufA*_::*recA*, that showed *sdrM* amplification, putative prophage induction, or mutations in the indicated loci. (C) Maximum DLX concentration that showed growth at each passage (shown as the respective number of generations) for the 12 independently evolved populations of the indicated strains. Orange lines represent populations that showed *sdrM* amplification, while grey lines represent populations without *sdrM* amplification. (D) The relative carrying capacity (normalized to the carrying capacity of the no DLX control condition), which represents the maximum value of reporter induction (normalized fluorescence), of the *recA* promoter reporter is shown for both the WT and the Δ*xerC* mutant at DLX concentrations lower than the respective IC-40 where the strains showed robust growth. The rate of increase (k) of the relative carrying capacity is similar for both strains. The carrying capacity was determined from the induction curves shown in S12C Fig using Growthcurver [44] for fitting and analysis. Data shown are the mean ± standard deviation of four independent biological replicates.

To further investigate the role of XerC, we tested multiple hypotheses for the amplification defect in the *xerC*::Tn mutant. We first measured the growth characteristics of the *xerC*::Tn mutant and found that, similar to several other mutants that had low DLX MICs (S1 Fig), the *xerC*::Tn mutant had a slower growth rate compared to the WT even in the absence of DLX (Fig 7A). However, given that we saw prevalent *sdrM* amplification in all the other slow-growing mutants tested (*rexB*::Tn, *recG*::Tn, and *recF*::Tn), a reduced growth rate does not correlate with a deficiency in gene amplification.

Next, to eliminate any polar effects of the *xerC*::Tn mutation, we generated a clean deletion mutant Δ*xerC*. The Δ*xerC* mutant had a similar growth rate to the *xerC*::Tn mutant, which could be complemented with a XerC expressing plasmid (S11A Fig). Further, complementation also reversed the DLX sensitivity of the Δ*xerC* mutant (S11B Fig), indicating that both the growth defect and DLX hyper-sensitivity of the Δ*xerC* mutant were due to the lack of functional XerC. To directly test whether XerC was important for gene amplification, we repeated our deep-well evolution with the Δ*xerC* mutant containing either an empty vector, or one expressing *xerC*. As a control, we also evolved the *recA*::Tn mutant containing either an empty vector, or one expressing *recA*. Given the slower growth rates of both the *recA*::Tn (Fig 3D) and Δ*xerC* (S11A Fig) mutants, we allowed each passage to grow for 48 hours (instead of the 24 hours we had done previously). Similar to what we observed in our earlier experiments, none of the populations containing the empty vector for either mutant showed *sdrM* amplification, while upon complementation, 9 out of 12 *recA*::Tn populations, and 7 out of 12 Δ*xerC* populations amplified *sdrM* (Fig 7B, C). These results further validate that RecA and XerC are important for *sdrM* gene amplification, and that the amplification defect seen in both mutants is likely not due to their slower growth rates.

A recent study suggested that the *xerC*::Tn mutant shows lower levels of *recA* expression and SOS response induction upon exposure to multiple DNA damaging agents [45]. Given the requirement of RecA for *sdrM* amplification upon DLX exposure, we hypothesized that the amplification defect in the *xerC* mutants could be due to lower expression of *recA*. Thus, we tested fluorescence of a *recA*::dsRed promoter reporter in the WT and Δ*xerC* backgrounds upon exposure to DLX. Given the higher DLX sensitivity of a Δ*xerC* mutant, we first established the respective DLX concentrations that had similar growth inhibitory effects for both strains (see Methods for details). For each strain, we determined the time at which the strain first reached maximum growth in the absence of DLX. We then identified the lowest DLX concentration where the growth (OD_600_) at that time point was below 60% of the maximum growth (inhibited by at least 40%) seen in the absence of DLX. We define this as the DLX IC-40 (Inhibitory concentration of 40%) for the respective strain (S12A, B Fig).

We tested the *recA* promoter reporter at 2-fold DLX concentrations (from 0.5x to 0.0675x the respective IC-40) and found that the induction of the reporter was similar for both strains at most concentrations, with the Δ*xerC* mutant showing slightly higher induction at the lower DLX concentrations (S12C Fig). At the highest DLX concentration tested (0.5x the respective IC-40), the WT had a significantly higher induction compared to the Δ*xerC* mutant. However, the growth of the Δ*xerC* mutant was considerably inhibited at this DLX concentration (S12B Fig), which may lead to non-specific defects in gene expression. Further, the normalized rates of induction of *recA,* as measured by the slope of the fit for the relative carrying capacity of the induction (normalized to the no treatment condition) over the DLX concentration, were similar for both the WT and the Δ*xerC* mutant (Fig 7D). Thus, in our experimental conditions, *recA* promoter induction is not significantly inhibited in a Δ*xerC* mutant upon exposure to DLX.

To further test whether the defect in *sdrM* amplification seen in the Δ*xerC* mutant was due to a deficiency in *recA* expression, we introduced plasmids expressing *recA* either under its native promoter or under the constitutive *tufA* promoter [46] into the Δ*xerC* mutant. Overexpression of *recA* under either of these promoters increased DLX MICs back to WT levels in a *recA*::Tn mutant but had no effect on DLX MICs in the Δ*xerC* mutant, indicating that the DLX sensitivity of the Δ*xerC* mutant cannot be compensated by RecA overexpression (S12D Fig). Further, we repeated our deep-well evolution with a Δ*xerC* mutant strain containing a plasmid expressing *recA* under the *tufA* promoter (to bypass any native *recA* regulation). None of the evolved populations showed *sdrM* gene amplification, indicating that the amplification defect in a Δ*xerC* mutant is independent of *recA* levels, and cannot be reversed by *recA* overexpression (Fig 7B, C).

## Discussion

**Fig 8:**
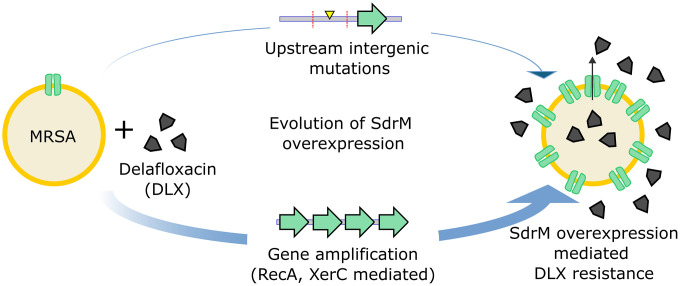
Overexpression of *sdrM* is a critical determinant of DLX resistance in *S. aureus.* Upon exposure to increasing concentrations of DLX, *S. aureus* evolves DLX resistance via overexpression of the gene encoding the SdrM efflux pump. This results either from frequent gene amplification of *sdrM* mediated by the RecA and XerC recombinases, or due to less frequent intergenic point mutations upstream of *sdrM*. Frequency of occurrence is denoted by the thickness of the arrows.

Gene amplification can lead to resistance against many distinct antibiotics in *S. aureus* [8], but modulators of amplification in *S. aureus* have not been described. In this study we used adaptive evolution to investigate the genetic effectors involved in *sdrM* gene amplification, and the selection and maintenance of amplified segments, that lead to DLX resistance in MRSA (Fig 8). Similar to other bacterial species, we determined that functional RecA was required for *sdrM* gene amplification in *S. aureus*. Given the lack of homology between the ends of amplified fragments, it is likely that RecA is not required for the initial duplication, but for the subsequent steps of higher order amplification. Additionally, we found that RecA was also involved in the expansion and contraction of amplified segments (Fig 3B and 3C). Stable amplified copies of *sdrM* in the *recA*::Tn background led to a growth defect (Fig 3D, S4 Fig), indicating that in the absence of the selective pressure of DLX, maintenance of amplified *sdrM* has a fitness cost, at least without functional RecA. We also found that mutants in other genes and pathways required for amplification in *E. coli* still showed *sdrM* amplification indicating that *S. aureus* may have alternate or redundant mechanisms for gene amplification. Finally, we identified that XerC, which has not been previously implicated in gene amplification, is a novel effector of gene amplification in MRSA, as mutants lacking functional XerC showed reduced rates of *sdrM* amplification and evolution of DLX resistance.

Mutations in several DNA repair-associated genes were observed when the *recA*::Tn strain was evolved both in the initial tube evolution (Fig 1A) and in the deep well evolution (Fig 7B). The *rexB* gene, which encodes a subunit of the AddAB helicase-nuclease that functions with RecA in DSB repair [23], was mutated almost exclusively in the *recA*::Tn background. While the effect of the specific RexB mutations will be the subject of future studies, it is possible that the mutations seen lead to altered enzyme activity that provide a selective advantage only when DSB repair is impaired in the absence of RecA.

Mutations in *recD2*, which encodes a helicase that has been implicated in various DNA repair functions in other species [30–32], were observed almost exclusively in mutants lacking *recA*, *rexA*, or *rexB* (S1, S6, S8 Tables), all of which are involved in DSB repair. Given the diversity of mutations seen in *recD2*, several of which are frameshift mutations, it is likely that the *recD2* mutations seen may be loss of function alleles. The specific role of RecD2 in *S. aureus* has not been characterized, but our data suggest that while the normal function of RecD2 may be in DNA repair, upon exposure to DLX, RecD2 activity may have a fitness cost in the absence of DSB repair. This is supported by the fact that a *recD2*^*A227E*^ allele leads to DLX hypersensitivity in the WT background (Fig 2C) but alleviates the sensitivity to DLX and other DNA damaging agents in the absence of a functional RecA (Fig 2D, E).

Another common mutation seen in the early steps of the *recA*::Tn evolution, was the intergenic mutation located in the predicted LexA binding site [25] between the *B7H15_RS12090* and *B7H15_RS12095* genes (P_*090/095*_) (Figs 1A, 2B). Three of the 16 previously reported SOS regulon genes in *S. aureus* are thought to be controlled by that LexA binding site [25], and we see increased expression of those genes in the P_*090/095*_^G→A^ mutant (Fig 5E). While the function of these genes in DNA repair has not been investigated, and bioinformatic annotations do not reveal an obvious connection to DNA repair, their regulation by the SOS response, and reduced DNA damage sensitivity in a mutant that overexpresses these genes suggests a putative role in DNA repair. The P_*090/095*_^G→A^ mutant also showed elevated expression of *sdrM*, which was immediately downstream of the SOS operon. Further work is necessary to determine whether *sdrM* is in the SOS regulon, and whether the transcriptional overexpression seen in this mutant is only due to disruption of LexA binding. In a previous study, a mutation in the same LexA-binding site was seen in isolates of the *S. aureus* COL strain that were selected for resistance against oxadiazoles, a new class of β-lactams [47]. SOS induction has been reported in *S. aureus* [33] and other species [48] upon exposure to β-lactams, indicating a role of this mutation and the mis-regulated genes in adaptation against diverse stresses.

In our evolved populations, we observed a negative association between *sdrM* amplification, and the LexA-binding site mutation or mutations in the upstream intergenic region of *sdrM*, both of which increase *sdrM* expression (Fig 5C). Further, a mutant carrying the P_*090/095*_^G→A^ mutation showed only a transient amplification in one out of three populations upon selection for DLX resistance (Fig 5D). Such transient amplification was rare in most other strains where amplified *sdrM* copies became fixed in the population once they arose (S6 Table), indicating that *sdrM* amplification may not have a significant selective advantage when *sdrM* is already overexpressed. Further, while complementing RecA back to the parental *recA*::Tn mutant led to an amplification frequency similar to that seen for the WT (Fig 7B, C), complementing RecA to the *recA*::Tn intermediate isolate R1 did not lead to *sdrM* amplification in two of the three evolved populations (Fig 1D). This is likely because R1 had the P_*090/095*_^G→A^ mutation and already expressed *sdrM* at a higher level. Thus, these *sdrM* overexpressing mutations represent an alternate route of DLX resistance to *sdrM* amplification. Notably, both these evolutionary trajectories of DLX resistance center around increased *sdrM* expression, underscoring the critical role of *sdrM* function in DLX resistance.

Of the 126 populations that were evolved for DLX resistance (Fig 5A), 108 populations showed gene amplification, while only 13 populations had at least one of the *sdrM* upstream or P_*090/095*_ mutations (of which 2 also showed *sdrM* amplification). Thus, *sdrM* amplification is selected at a significantly higher frequency compared to the specific point mutations, consistent with what has been reported before for the relative frequencies of these two types of mutations [7].

The varied amplified *sdrM* fragments in our evolved populations showed limited homology between the fragment ends (Fig 4E). Similar lack of homology in amplified fragments has been reported in other species such as *Acinetobacter* sp., *E. coli*, and *Salmonella*, upon exposure to diverse selection pressures [18,19,49]. Almost all of our amplified fragments had one end nearly 4 kb downstream of *sdrM* in the tRNA-rRNA loci, while the other end was in a region starting immediately upstream of *sdrM*. This ~4 kb region contains four genes – *lmrS* and *sepA,* encoding two other efflux pumps, and genes encoding a P-loop NTPase, and a 61 amino acid hypothetical protein. We had previously shown that neither *lmrS* or *sepA*, nor a combination of all the four genes contributes to DLX resistance [16]. Further, here we show that mutants with transposon insertions in *sepA* or the P-loop NTPase encoding gene still show prevalent gene amplification (Fig 5A, 6A-C).These data indicate that amplification of this region is selected solely due to the presence of *sdrM*, and that the amplified fragments terminating in the tRNA-rRNA loci, and thus including the ~ 4 kb region, is a property of the amplification process. We postulate there are two, non-exclusive underlying reasons for this observation. First, amplified fragment ends in the tRNA-rRNA locus could have a higher selective advantage as amplified copies of this locus would lie upstream of *sdrM* and could lead to higher *sdrM* expression due to potential read-through transcription, similar to what has been seen in *Streptococcus pneumoniae* [50]. Second, the highly transcribed tRNA-rRNA clusters are thought to be hotspots for codirectional replication-transcription conflicts [51], and any resulting DNA damage may lead to a higher prevalence of duplications ending in this cluster. Future studies will focus on the mechanistic investigation of these possibilities. The proximity of *sdrM* and its homologs to rRNA-tRNA loci is conserved in many related species (S5 Fig), raising the possibility that amplification of these genes may be a phenomenon seen across species upon exposure to the appropriate selective pressures.

Almost all our evolved populations showed increased coverage of an intact prophage (Fig 5A, S8 Fig), likely indicating prophage induction due to DNA damage [52]. This prophage encodes a homolog of RecT, that has been implicated in recombination without homology or between short homologous sequences [53,54]. However, we did not detect significant prophage induction in a *recT*::Tn mutant (Fig 5A), but still saw *sdrM* amplification, indicating that while RecT likely plays a role in the induction of this prophage, *sdrM* amplification can occur in the absence of RecT and prophage induction. Further, there were multiple populations, e.g., in the *recX*::Tn, *rarA*::Tn, and *ruvX*::Tn mutants (S6 Table), where we saw prophage induction, but no *sdrM* amplification, suggesting these two events are unlinked.

In our broad screen for genetic determinants of gene amplification, of the 40 transposon mutants we evolved, only *recA*::Tn and *xerC*::Tn were significantly impaired in *sdrM* gene amplification (or maintenance or selection of amplified segments). DNA repair pathways in *S. aureus* have been only partly characterized, and the roles of several DNA repair proteins as well as redundancies present in various repair pathways have not been fully dissected [23,39]. The list of screened mutants included those with transposon insertions in genes involved in DSB repair, nucleotide excision repair, base excision repair, Holliday junction resolution, and cell division (S3 Table) [23,39]. In *E. coli*, it has been shown that mutants in DSB repair (*recA*, *recB*, *ruvC*) are necessary for amplification [19], while in *Salmonella enterica* Typhimurium*,* individual mutations in *recB* and *recF* did not significantly alter the frequency of gene duplication on a plasmid (higher order amplification was not tested), while a *recA* mutant and a *recB recF* double mutant showed significantly lower rates of duplication [18]. Our data show that unlike *E. coli*, mutants in RexA or RexB, which are components of the RexAB (AddAB) helicase/nuclease that performs a similar function as RecBCD in DSB repair, still show gene amplification. Given the requirement of RecA, this indicates that other helicase/nuclease combinations may function with RecA in homologous recombination during amplification. Further, while DNA polymerase I (PolA) is required for amplification in *E. coli* [19,55], the *S. aureus polA*::Tn mutant showed prevalent amplification. Similar to *E. coli* [19], mutations in DNA polymerases IV and V (DinB and UmuC) still allowed for amplification, as did mutations in another DNA polymerase encoded by *RS08105* indicating that in *S. aureus* other polymerases may play a role in amplification, or there may be redundancies in polymerase function. Thus, gene amplification in *S. aureus* does not require DSB repair and is likely mediated by novel determinants.

One such determinant that we identified is the tyrosine recombinase XerC. We found that the lack of a functional XerC significantly reduced the frequency of *sdrM* amplification. XerC is a widely conserved protein involved in the segregation of daughter chromosomes during cell division, where it functions with the related recombinase XerD to bind *dif* sites near the chromosomal terminus, and with the DNA translocase FtsK [41,56]. In *S. aureus*, the *xerC*::Tn mutant has increased sensitivity to DNA gyrase inhibiting and cell-wall synthesis targeting antibiotics, and was reported to be deficient in the induction of *recA* [45]. However, any role of XerC in gene amplification remains unknown. The *S. aureus* chromosomal terminus is located around the genomic coordinate ~1.47 Mb [57], while *sdrM* is located around ~2.30 Mb, indicating that the *S. aureus dif* sites are not located in the vicinity of *sdrM*. However, the presence of cryptic *dif*-like or other XerC target sites near the *sdrM* locus cannot be ruled out and will be explored further. In *S. aureus*, XerD is an essential protein, unlike in other species, suggesting that it may have additional functions [56]. We found that the Δ*xerC* mutant did not have a defect in *recA* induction upon exposure to DLX in our experimental conditions, and that overexpression of RecA did not complement the Δ*xerC* defect in gene amplification. Further, mutants in other genes that are critical for chromosomal separation, *noc*::Tn and *scpB*::Tn [58–60], still showed prevalent amplification (Fig 5A), indicating that a defect in chromosomal separation was not sufficient to inhibit gene amplification. Thus, XerC plays an unknown, likely RecA-independent, role in gene amplification, possibly via promoting recombination or resolution of recombination intermediates which may be mediated by cryptic *dif*-like or other XerC target sites, and future work will investigate this mechanism further.

In our tube evolutions, one out of the three *xerC*::Tn populations amplified *sdrM* albeit in the later passages (S6 Table), while none of the 30 total populations of the *xerC*::Tn or Δ*xerC* mutants evolved in deep-well plates showed *sdrM* amplification. This is likely due to the difference in selective pressures in the two evolution schemes. In the tube evolution, populations are passaged in increasing concentrations of DLX, allowing for longer times for growth if required, leading to a high selective advantage of, and thus the selection of, DLX resistance mutations even if they arise at a very low frequency or have significant fitness defects. However, in the deep-well evolution scheme, at every passage, populations are grown in a two-fold serial dilution series of DLX and can thus continue to be passaged at the same DLX concentration without gaining additional DLX resistance. Further, due to the higher media volume in the tubes, population sizes are larger in the tube evolution, possibly allowing for the selection of highly infrequent mutation or amplification events.

Gene amplification can lead to rapid evolution of resistance against many classes of antibiotics, including ones that have multiple targets in the cell [8,16]. Pathways required for such amplification can thus be putative targets in combination with antibiotics, to lower the frequency of resistance evolution. We show that evolution of DLX resistance is inhibited in *S. aureus* in the absence of XerC, and further work is required to evaluate its potential as a therapeutic target. Additionally, our data suggest that, apart from RecA and XerC, genes with redundant function may play a role in gene amplification in *S. aureus*, and that such genes and pathways may differ between bacterial species. Combinatorial mutagenesis, and genome-wide screens may thus be critical to identify additional determinants of gene amplification.

## Methods

### Strains and growth conditions

All strains and plasmids used in this study are listed in S9 Table.

For experiments in liquid media, bacteria were grown at 37^o^C, shaking at 300 rpm in modified M63 media (13.6 g/L KH_2_PO_4_, 2g/L (NH_4_)_2_SO_4_, 0.4 μM ferric citrate, 1mM MgSO_4_; pH adjusted to 7.0 with KOH) supplemented with 0.3% glucose, 0.1 μg/mL biotin, 2 μg/mL nicotinic acid, 1 × Supplement EZ (Teknova) and 1 × ACGU solution (Teknova) [16]. For experiments with 96-well or deep well plates, the plates were incubated in a Titramax 1000 (Heidolph) incubator at 37^o^C shaking at 900 rpm. To construct and mutate plasmids and strains, cells were grown in LB liquid medium (10 g/L bacto-tryptone, 5 g/L yeast extract, 10 g/L NaCl) at 25^o^C, 30^o^C, or 37^o^C as indicated, shaking at 300 rpm, or on LB plates (15 g/L agar) supplemented with the appropriate antibiotics (10 μg/mL chloramphenicol, 50 μg/mL kanamycin, 10 μg/mL trimethoprim, 10 μg/mL erythromycin) or 0.4% para-chlorophenylalanine (PCPA).

### Minimum inhibitory concentration measurements

MICs were tested as described previously in modified M63 media [16]. Briefly, a 96-well flat clear bottom plate (Corning) was used to prepare two-fold serial dilutions with eight concentrations of DLX. Cells grown overnight in modified M63 were diluted 1:5000 in fresh modified M63 and added at a 1:1 ratio to the 96-well plate with the DLX dilutions such that the final dilution of the cells was 1:10000. Cells were grown for 24 hours in a Titramax 1000 (Heidolph) incubator at 37^o^C, shaking at 900 rpm. The *recA*::Tn, 1.7a *recA*::Tn, *rexA*::Tn, and *xerC*::Tn strains in Fig 3A and S1 Fig, were grown for an additional 24 hours to compensate for their slower growth. Following the growth, OD_600_ was measured using a microplate reader (Biotek Synergy H1) and the MIC values were determined by fitting the OD_600_ vs. DLX concentration to a modified Gompertz function [61].

### Growth curves and analysis

Cells were grown overnight in tubes with 2 mL of modified M63, diluted 1:100 in 100μL or 200μL modified M63 and added to a COSTAR clear bottom 96-well plate. OD_600_ was measured using a microplate reader (Biotek Synergy H1) every 30 min for the indicated time, while shaking at 800 rpm at 37°C. The growth curves were analyzed with the R package Growthcurver to determine growth rate, doubling time, the time to reach the maximum growth rate, and carrying capacity [44].

### Experimental evolution of DLX resistance in tubes

Evolution of *S. aureus* JE2 cells in tubes was carried out as described previously [16]. Briefly, for most strains, three independent colonies were picked and cultured in 2 mL of modified M63. After overnight growth, cells were transferred to new tubes containing 2 mL modified M63 with DLX at a concentration ~ 0.5x the respective MIC. After 24 h growth, 40 μL cells were transferred to tubes containing twice the concentration of DLX as the prior passage in 2 mL modified M63 and this was repeated up to a DLX concentration of 2 μg/mL. If sufficient growth was not visually apparent after 24 hours, cells were given an additional 24–48 hours to grow. If growth was still not seen, the evolution was reset to the previous passage and the DLX increment was adjusted depending on the growth. If gene amplification was not detected in the final passage (2 μg/mL), evolutions were continued similarly and carried out until a terminal concentration of 8 or 16 μg/mL.

For mutants (*xerC*::Tn and *recG*::Tn) which had very low MICs (<10-fold the WT MIC), a single population was evolved until the DLX concentration in the evolution approached the JE2 WT MIC (~0.22 μg/mL). For the next passage, cells were cultured with a dilution of 1:50 into three independent tubes containing twice the previous DLX concentration, and subsequently these three populations were independently evolved as mentioned above. For the *recA*::Tn mutant, we sequenced intermittent passages beginning from those that grew in a DLX concentration one-tenth of the WT MIC, i.e., 0.025 µg/mL DLX. For the other tube evolutions, we sequenced the passage that grew at 2 µg/mL, and if subsequent evolutions were performed, we sequenced intermittent passages, as well as the terminal one.

### Experimental deep well evolution of DLX resistance

Mutants that needed to be tested further for amplification frequency were streaked out on selective plates, and 12 independent colonies per strain were cultured in 400 μL of modified M63 in deep well plates (Nunc 96 DeepWell Polystyrene Plates) for 24 h with shaking at 37^o^C. For the *xerC*::Tn mutant, the original evolved population (prior to splitting the evolution into three independent populations as described above), that had an MIC similar to the WT, was streaked out on a plate. A single colony (XC5_7) was re-streaked out, and 12 colonies from this plate were cultured to start the 12 independent populations. 4 μL cells were then transferred to a fresh deep well plate containing 400 μL of modified M63 with 8 serially diluted DLX concentrations, where the maximum DLX concentration was set at 1–2 μg/mL for the data shown in Fig 6A-C, and for the complemented versions of *recA*::Tn and Δ*xerC* in Fig 7B, C, and at 0.03125-0.125 μg/mL for the other strains shown in Fig 7B, C. This meant that each independent population was cultured in all the 8 DLX concentrations. For the subsequent passages, 8μL of cells in the well at the highest DLX concentration that showed growth (defined by OD_600_ > 0.400 as measured in a Biotek Synergy H1 microplate reader) were transferred into a new deep well plate containing the same DLX concentration profile and grown for 24 h. The maximum DLX concentration for the subsequent passages was increased if there was growth in the 2^nd^ well of the previous passage. The evolution was continued for 14 passages in total for the strains in Fig 6A-C and 11 passages for the strains in Fig 7B,C, and the genomic DNA of the terminal passage was sent out for whole genome sequencing.

### Whole genome sequencing

Genomic DNA was extracted from cultures using the Qiagen DNeasy Blood and Tissue kit. Sequencing libraries were prepared using the Illumina Nextera XT DNA Library Preparation kit and sequenced (75 cycle or 100 cycle) on the Illumina NextSeq 550 (75 cycle) or the NextSeq 2000 (100 cycle) instruments to obtain single-end reads. The FASTQ files were processed by removing adapters and trimming using fastp v0.23.2. Sequences were aligned, variants called, and novel junctions identified using breseq v0.37.17 [62]. The mutations were marked as present in the respective figures (Figs 1A, 1D, 5A-D) if at least 30% of the population contained the particular mutation. Copy number variation in the efflux pump *sdrM* was identified as described previously [16]. Briefly, we used the BAM2COV command in breseq to determine the read coverage depth across the whole genome. The *sdrM* copy number was determined by dividing the average coverage depth of *sdrM* with the average coverage depth of the whole genome (both normalized to length). The threshold used to define *sdrM* amplification was the presence of a novel junction around *sdrM* and an *sdrM* copy number greater than 1.3x the WT *sdrM* copy number. Gene maps shown in Fig 4 and S7 Fig were drawn using *gggenes* [63].

### Construction of allele replacement, overexpression, and reporter strains in *S. aureus* JE2

Construction of the allele replacement mutants and overexpression strains in JE2 was done as described previously [16]. Briefly, for the allele replacement, the new allele with flanking homology, or upstream and downstream regions with flanking homology for the Δ*xerC* mutant, were cloned into the pIMAY* plasmid [64], all of which had been PCR amplified. The plasmid was then electroporated into *S. aureus* JE2. Strains carrying the integrated plasmid were selected at 37^o^C (the selective temperature), the plasmids were subsequently excised via PCPA counter-selection, and plasmid loss was confirmed via testing for chloramphenicol sensitivity. The final mutant was confirmed by flanking PCR and Sanger sequencing.

To construct the overexpression strains, the appropriate genes with their native promoters were cloned into the pKK30 vector [65] and electroporated into *S. aureus* JE2. To bypass the native regulation of *recA* and constitutively express it, *recA* was expressed from a *tufA* promoter [46] fused to the *gyrA* leader sequence. A gene fragment containing the *tufA* promoter (289 bp region ending 45 bp upstream of the start codon) fused to the regulatory region of *gyrA* (38 bp immediately upstream of the start codon) was ordered from IDT (sequence provided by the lab of Shaun Brinsmade, Georgetown University). The native *recA* promoter from pKK30-*recA* was replaced by this gene fragment.

To construct pKK15A, the backbone was derived from pKK30 [65], removing the native R6K origin and the P_*sarA1*_::*dfrA* (trimethoprim resistance marker). The kanamycin resistance cassette (*aphA3*) was derived from the pKAN plasmid from the NTML library toolbox [66]. The p15A origin was derived from pIMAY* [64]. Column-purified PCR fragments were concatenated using a three-fragment Gibson assembly and transformed into *E. coli* IM08B to generate pKK15A. The P_*sarA1*_::*dsRed3.T3* construct was amplified from pKM16 [67] and inserted into pKK15A to generate pKK15A P_*sarA1*_::*dsRed3.T3*, and P_*sarA1*_ was subsequently replaced by P_*recA*_ to generate the *recA* promoter reporter (pKK15A P_*recA*_::*dsRed3.T3*).

Primers used for constructing all mutants and overexpression strains are listed in S10 Table.

### Mutagenesis of *lexA* gene

The Agilent QuikChange Lightning Site-Directed Mutagenesis Kit was used as per the manufacturer’s instructions to generate the *lexA* mutant alleles. Mutagenic primers were created utilizing the Agilent web-based QuikChange Primer Design tool and are listed in S10 Table.

### Phage transduction

The donor strains (*recA*::Tn or *sdrM*::Tn) were grown overnight in LB, diluted 100-fold in fresh LB the following day, and grown at 30^o^C for 1.5-2 h. Then, 1 mL of 10 mg/mL CaCl_2_ and 10 μL of 10^10^ pfu/mL bacteriophage 85 (phi85) suspension was added to this culture which was incubated at room temperature for 30 min without shaking and then rotated slowly (80 rpm) at 37^o^C for 5–6 h or overnight. Afterwards, the culture was filtered using a 0.45 μm filter and the phage-containing filtrate was stored at 4^o^C.

To transduce the transposon mutations to the appropriate JE2 backgrounds, we grew the recipient cells overnight in 2 mL LB, and collected the cell pellets the following day by centrifugation. Cell pellets were resuspended in 300 μL of LB broth with 5 mM CaCl_2_ and 700 μL of the donor phi85 phages were added. This mixture was incubated at 37^o^C without shaking for 20 min. Cells were then immediately washed two times with 1mL of 40 mM sodium citrate and finally plated on LB + erythromycin plates containing 200 μM sodium citrate. The transposon insertion was confirmed via PCR and in the case of the P_*090/095*_^G→A^ mutant with the *sdrM*::Tn mutation, a second PCR was conducted to confirm the presence of the P_*090/095*_^G→A^ mutation to rule out potential transduction-based replacement due to the proximity of this mutation to the chromosomal position of *sdrM*.

### Synteny analysis

The protein sequence of SdrM was analyzed by BLASTP [68] against the ClusteredNR database (nr_cluster_seq). The Constraint-based Multiple Alignment Tool was selected for the top 100 searches under default parameters, and for each strain cluster the top candidate with an assembled genome was chosen for the alignment. The corresponding genomes were identified, and using the NCBI nucleotide database, the distance between the closest rRNA/tRNA locus and the start codon of *sdrM* was manually determined.

### Fluorescent promoter-reporter assay

Cells were grown overnight in LB media with 50 μg/mL kanamycin to maintain the pKK15a-dsRed plasmid. To minimize the formation of cell clumps that will disrupt the fluorescent readings LB media was chosen. The following day an 8-well 2-fold dilution series of DLX was made in LB + Kanamycin, in a 96-well Black clear flat bottom plate (COSTAR), and blank controls were also included. Overnight cells were diluted 1:100 into this plate and grown in a microplate reader (Synergy) at 37^o^C for 24 hours. At each 20-minute interval the fluorescence (excitation 560 nm and emission 587 nm) and optical density (600nm) were measured. The first 200 minutes of the data points were discarded because of high noise due to lag phase growth. The growth for each concentration tested for each mutant was fitted to a non-logistic curve using the R package Growthcurver [44]. The fitted values for the carrying capacity were averaged across the four independent biological replicates for the no-DLX condition (for WT and the Δ*xerC* mutant) and the time at which the optical density of the mean growth for the no-DLX condition reached the mean carrying capacity was determined. We set a threshold to define robust growth as an OD_600_ value at this time-point that was ≥ 60% of the mean carrying capacity for the no-DLX condition. The lowest DLX concentration that inhibited growth more than 40% at this time point was defined as the IC-40, i.e., the lowest DLX concentration whose OD_600_ at this time-point was less than the threshold. All treatment conditions that showed a higher optical density than the threshold at this time-point were deemed as having robust growth and considered for subsequent analysis.

The ratio of the background subtracted fluorescence to the corresponding optical density at each time-point was defined as the normalized fluorescence. The average normalized fluorescence for all the selected DLX concentrations and the no-DLX condition across all four independent biological replicates were fit to logistical growth curves using the R package Growthcurver [44], and the average carrying capacity for each condition, which signifies the maximum normalized fluorescence reached, was determined. To compare the two strains directly, the relative carrying capacity was determined where the carrying capacity for each DLX concentration was normalized to the respective no-DLX control. The slope (k) of the datapoints in Fig 7D was calculated by a linear fit of the relative carrying capacity of the normalized fluorescence using the exponential growth with log(population) equation for nonlinear regression analysis in GraphPad Prism.

### Quantitative PCR (qPCR)

For quantifying *sdrM* copy number, qPCR was done as described previously [16]. Briefly, gDNA samples diluted to 10 ng/µL were mixed with appropriate qPCR primers and Applied Biosystems Power SYBR Green PCR Master Mix (Thermo Scientific) in a Microamp EnduraPlate Optical 384 Well Clear Reaction Plate (Thermo Fisher Scientific) and the qPCR was carried out in a QuantStudio 5 real-time PCR machine (Thermo Fisher Scientific). We used *rpoC* as the control housekeeping gene. To determine the limit of detection for the junctions, 1 μL gDNA of the evolved population (10 ng/µL) was serially diluted 10-fold in 9 μL of genomic DNA from the parental WT strain (10 ng/µL) seven consecutive times. Then the qPCR was carried out as described above.

### Reverse transcription quantitative PCR (RT-qPCR)

To determine the expression level of genes neighboring the *lexA* binding site including *sdrM*, cells grown overnight in tubes with 2 mL modified M63 were diluted 1:100 in 10 mL of modified M63 in a flask and grown for an additional 3–4 hours to an OD_600_ of 0.3-0.4. For the *sdrM* allele-replacement strains, we similarly grew the cells to an OD_600_ of ~0.5 and also took a sample of overnight grown cells. 2x volume of RNAprotect Bacteria Reagent (QIAGEN) was added to the culture and after 10 min incubation at room temperature, cells were centrifuged at 4000 rpm, the supernatant removed, and the pellets stored at -80°C. RNA was extracted using the NORGEN BIOTEK Total RNA purification kit and genomic DNA was removed with the TURBO DNA-free Kit (Invitrogen). Absence of genomic DNA was confirmed via PCR. cDNA was synthesized utilizing the Superscript III Reverse Transcriptase (Thermo Fisher Scientific) with random primers. The subsequent qPCR was carried out as described in the previous section.

### TOPO TA cloning

Genomic DNA used as a template for the PCR reactions was concentrated to ~200 ng/µL and 2 µL of it was added to each 23 µL PCR reaction. PCR reactions were performed using the OneTaq or LongAmp polymerases, purified with the Zymo Clean and Concentrator-5 kit, and cloned into the pCR4-TOPO TA plasmid using the TOPO TA cloning kit for Sequencing, with One Shot TOP10 Chemically Competent *E. coli* (Invitrogen). Blue-white selection was used to identify colonies carrying plasmids with inserts by supplementing the LB + Kanamycin plates with 40 μg/mL of X-gal (Invitrogen). Plasmids were extracted using the Qiaprep Spin Miniprep kit (Qiagen) and sequenced via whole plasmid sequencing to identify the inserted fragments.

### Determination of amplification stability

For the amplification stability experiment with 1.7a (*recA*::Tn), cells were inoculated into modified M63 from the corresponding frozen stock and grown overnight. For the amplification loss experiment, which was done for five passages, cells were grown from frozen stocks into modified M63 containing DLX at ~0.25x the corresponding MICs, in order to stabilize the amplified fragments prior to the treatment-free passaging. The cultures were then diluted 1:1000 in 2 mL of modified M63 (with or without DLX) and grown for 24 h. Genomic DNA was extracted from the remaining culture. This procedure was repeated for the appropriate numbers of passages, and WGS was performed on the genomic DNA from each passage. Copy number for *sdrM* was determined as described above.

### TUNEL assay

We followed the terminal deoxynucleotide transferase dUTP nick end labeling (TUNEL) assay as described previously for bacteria [69]. Briefly, cells were grown overnight in 2 mL of modified M63, diluted 1:50 in 15 mL of modified M63, and grown to an OD_600_ of 0.3-0.4. Next, 2 mL of cells were transferred to three falcon tubes, and two of the tubes were treated with the appropriate concentration of DLX or MMC respectively. The remaining tube was kept as a treatment-free control, and all three tubes were grown for an additional 3 h. From these cultures, 1 mL was transferred to microcentrifuge tubes, washed once with ice cold 1x Phosphate buffered saline (PBS,1X, Thermo Scientific), and fixed by treatment with 1mL of 4% Paraformaldehyde (PFA) on ice for 30 min. Cells were then washed with ice cold PBS once, and resuspended in 250 μL of PBS and 1mL of ice cold 70% ethanol, and incubated 12–16 hours at -20^o^C.

After incubation, the tubes were centrifuged, the supernatants removed, and the cells labeled with the Apo-Direct Kit (BD Bioscience). Fluorescence output was analyzed using an Apogee MicroPLUS flow cytometer (ApogeeFlow Systems Inc), with excitation and emission wavelengths of 488 nm and 515 nm respectively. A region of interest (ROI) was drawn around the WT cell events for medium vs large light scatter angle, and this ROI was used as a gate for subsequent analysis of other strains. The proportion of the population with DNA damage was quantified as the Overton positive percentage using FlowJo (v10.1) [70].

### Statistics

Statistical comparison of datasets was performed using GraphPad Prism 9. Tests used and significance thresholds are mentioned in the respective figure legends.

## Supporting information

S1 Fig Selected mutants show a wide range of DLX sensitivities. DLX MICs of WT and the indicated mutants were measured, and the mutant MICs were normalized to the WT MIC. Data shown are the mean ± standard deviation for three independent biological replicates. Significance is shown for comparison to a value of 1, as tested by one-sample *t-*tests (* *p* < 0.05, ** *p* < 0.01, *** *p* < 0.001, # *p <* 0.0001).
(TIF)

S2 Fig DLX causes DNA damage. (A) Representative fluorescence from TUNEL staining of WT cells treated with no antibiotic, 1 µg/mL MMC, or 5 µg/mL DLX, or a no-dye control. (B) The Overton positive percentage (representing the percentage of the population that has increased fluorescence compared to the control no antibiotic population) for the DLX and MMC treated samples. Significance is shown for comparison to a value of 0, as tested by one-sample *t-*tests (* *p* < 0.05).
(TIFF)

S3 Fig Mutations seen in RexB and RecD2 in the initial *recA*::Tn evolved populations are in relatively conserved residues. (A, B) Multiple sequence alignments of (A) RexB and (B) RecD2 from the Conserved Domain Database [71,72], where residues in red are highly conserved, and those in blue are not. Arrows denote the position of mutations, which are also highlighted, seen in the initial *recA*::Tn evolved populations.
(TIFF)

S4 Fig Amplified segments containing *sdrM* have a fitness cost. (A) Growth rates, (B) doubling times, and (C) time to reach the peak growth rate are shown for the WT, and 1.7a, and *recA*::Tn mutants in both backgrounds. These parameters were obtained from growth curves shown in Fig. 3D using Growthcurver [44] for fitting and analysis. Data shown are the means ± standard deviation for three independent biological replicates. Significance is shown for comparison to the 1.7a *recA*::Tn strain, as tested by a one-way ANOVA with Dunnett’s test for multiple comparisons (* *p* < 0.05, ** *p* < 0.01, # *p <* 0.0001).
(TIF)

S5 Fig Proximity of *sdrM* homologs to the rRNA-tRNA locus is conserved in related species. Homologs of *sdrM* were identified using BLASTP [68] against the ClusteredNR database on NCBI using default settings. Shown are the distances of the *sdrM* homologs to the nearest rRNA-tRNA cluster.
(TIF)

S6 Fig Novel junctions corresponding to the *sdrM* amplification can be detected at a 10^-4^ – 10^-5^ dilution. qPCR C_T_ values for the control housekeeping gene *rpoC* and the respective novel junction present in two different evolved DLX resistant populations from (A) WT, (B) *spoIIIE*::Tn, and (C) *recQ2*::Tn, measured in a 10-fold dilution series where genomic DNA from the evolved population was diluted in genomic DNA from the parental strain.
(TIF)

S7 Fig Rare novel junctions around *sdrM* may be present in the unselected WT strain. (A) Inverse-PCR like strategy to detect novel junctions present around *sdrM*. Shown are two hypothetical amplified fragments (outlined by the brown lines). We designed outward facing primers (Primer 1 and Primer 2) binding to *sdrM* and *B7H15_RS12060*. If a duplicated or amplified fragment is present in that region, the novel junction should get amplified by the PCR primers. (B) Three putative duplicated or amplified fragments identified in the WT strain using the inverse PCR like strategy.
(TIFF)

S8 Fig The second region with increased coverage in several DLX resistant evolved populations encodes for a putative intact prophage. (A) Genomic coverage from whole-genome sequencing of a WT evolved population showing increased coverage for the 43 kb prophage region as well as the *sdrM* locus. (B) The JE2 genome was analyzed using PHASTER [35] to identify prophage regions, and the PHASTER output is shown. Region 4 (in green) encodes an intact prophage whose coordinates match the region that shows increased sequencing coverage depth in the evolved DLX resistant populations.
(TIFF)

S9 Fig An intergenic mutation upstream of *sdrM* leads to increased *sdrM* expression. Expression of *sdrM* was measured in log phase (OD_600_ = 0.5) and stationary phase (overnight cultures) of the previously reported sdrM2* and sdrM3* allele-replacement mutants [16], using qPCR where the *rpoC* gene was used as the control housekeeping gene. The sdrM2* mutant has the A268S coding sequence mutation, while the sdrM3* mutant has the A268S mutation as well as a C to G change at position -164 (upstream of *sdrM*). Data shown are the means ± standard deviation for three independent biological replicates. Significance is shown for comparison to 1, as tested by a one-sample *t-*test (** *p* < 0.01).
(TIF)

S10 Fig Scheme for the deep well evolution assay. 12 independent populations of the strain to be evolved were inoculated in a 2-fold dilution series of DLX and grown for 24 or 48 hours depending on the specific strains being tested. Populations from the well with the highest DLX concentration that showed growth were transferred to the next passage. Wells showing growth are denoted in yellow, and of those, the ones in the highest DLX concentration are marked with the red circles. Populations from these red-circled wells were propagated to the next passage. After the specified number of passages, the terminal passages from the wells with the highest DLX concentration that showed growth were sent for WGS.
(TIFF)

S11 Fig Growth and DLX resistance defects in the Δ*xerC* mutant can be complemented by XerC expression. (A) Overnight cultures of the indicated strains were diluted 1:100 in modified M63 medium and OD_600_ was measured every 30 minutes. Data shown are mean ± SEM of three independent biological replicates. (B) DLX MICs of the Δ*xerC* mutant containing either the empty pKK30 plasmid or one expressing *xerC* from its native promoter. Data shown are mean ± standard deviation for three independent biological replicates. Significance is shown for comparison to the empty vector control, as tested by an unpaired *t-*test (# *p <* 0.0001).
(TIFF)

S12 Fig The Δ*xerC* mutant does not have a defect in *recA* induction upon DLX exposure. (A-C) WT and the Δ*xerC* mutant were grown in a dilution series of DLX, and fluorescence (excitation 560 nm; emission 587 nm) and A_600_ measured every 20 minutes. Shown are the mean ± SEM of three independent biological replicates. A_600_ of (A) WT and (B) the Δ*xerC* mutant is shown. The horizontal dotted line represents 60% of the mean carrying capacity for the no DLX condition, and the vertical dotted line represents the time at which the growth without DLX first reaches the mean carrying capacity. The growth curves in green represent the DLX concentrations below the respective IC-40, and the curves in grey represent the DLX concentrations equal to or greater than the respective IC-40. (C) Normalized fluorescence (relative to the OD_600_) for WT and the Δ*xerC* mutant grown in 0.5x, 0.25x, 0.125x, 0.0675x the respective IC-40, or without DLX. (A-C) Data shown are the mean ± SEM of four independent biological replicates. (D) DLX MICs of the WT, *recA*::Tn, and Δ*xerC* containing either the pKK30 empty vector, or one containing P_*recA*_::*recA* or P_*tufA*_::*recA* (for *recA*::Tn, and Δ*xerC*). Data shown are the mean ± standard deviation of three independent biological replicates. Significance is shown for comparison to the respective empty vector control, as tested by a one-way ANOVA followed by a Tukey’s test for multiple comparisons (# *p <* 0.0001).
(TIFF)

S1 Table Mutations seen in the *recA*::Tn evolved populations. (XLSX)

S2 Table Mutations seen in the evolved populations of *recA*::Tn intermediate isolate R1 complemented with either an empty pKK30 plasmid or one carrying *recA.* (XLSX)

S3 Table List of strains evolved for DLX resistance. (XLSX)

S4 Table The distinct amplified fragments observed in the evolved populations from the tube evolutions of transposon and allele-replacement mutants. (XLSX)

S5 Table Distances between *sdrM* homologs and the nearest rRNA locus in multiple *Staphylococcus* and *Mammaliicoccus* species. (XLSX)

S6 Table Mutations seen in the evolved populations from the tube evolutions of transposon and allele-replacement mutants. (XLSX)

S7 Table Mutations seen in the evolved populations of the P_*090/095*_^G→A^ allele-replacement strain. (XLSX)

S8 Table Mutations seen in the evolved populations from the deep-well evolutions. (XLSX)

S9 Table Strains and plasmids used in this study. (PDF)

S10 Table Primers used in this study. (PDF)

S1 Data Numerical data underlying graphs shown in figures. (XLSX)
