# Supplementary figures and images for: Modulators of gene amplification alter evolution of antibiotic resistance in Staphylococcus aureus
Source: PLoS Genet. 2025 Dec 31;21(12):e1012011. doi: 10.1371/journal.pgen.1012011 (PMC12795462; doi:10.1371/journal.pgen.1012011)

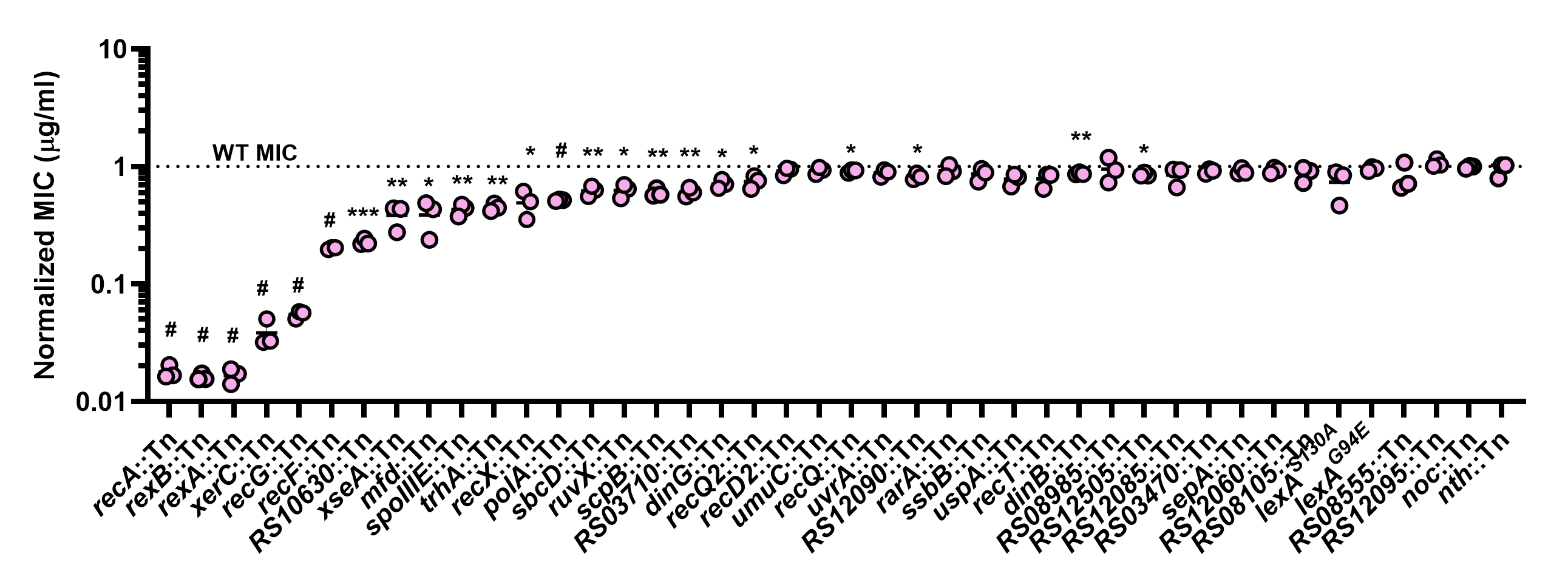

Supplement: S1 Fig — DLX MICs of WT and the indicated mutants were measured, and the mutant MICs were normalized to the WT MIC. Data shown are the mean ± standard deviation for three independent biological replicates. Significance is shown for comparison to a value of 1, as tested by one-sample t-tests (* p < 0.05, ** p < 0.01, *** p < 0.001, # p < 0.0001). (TIF) [file pgen.1012011.s001.tif]

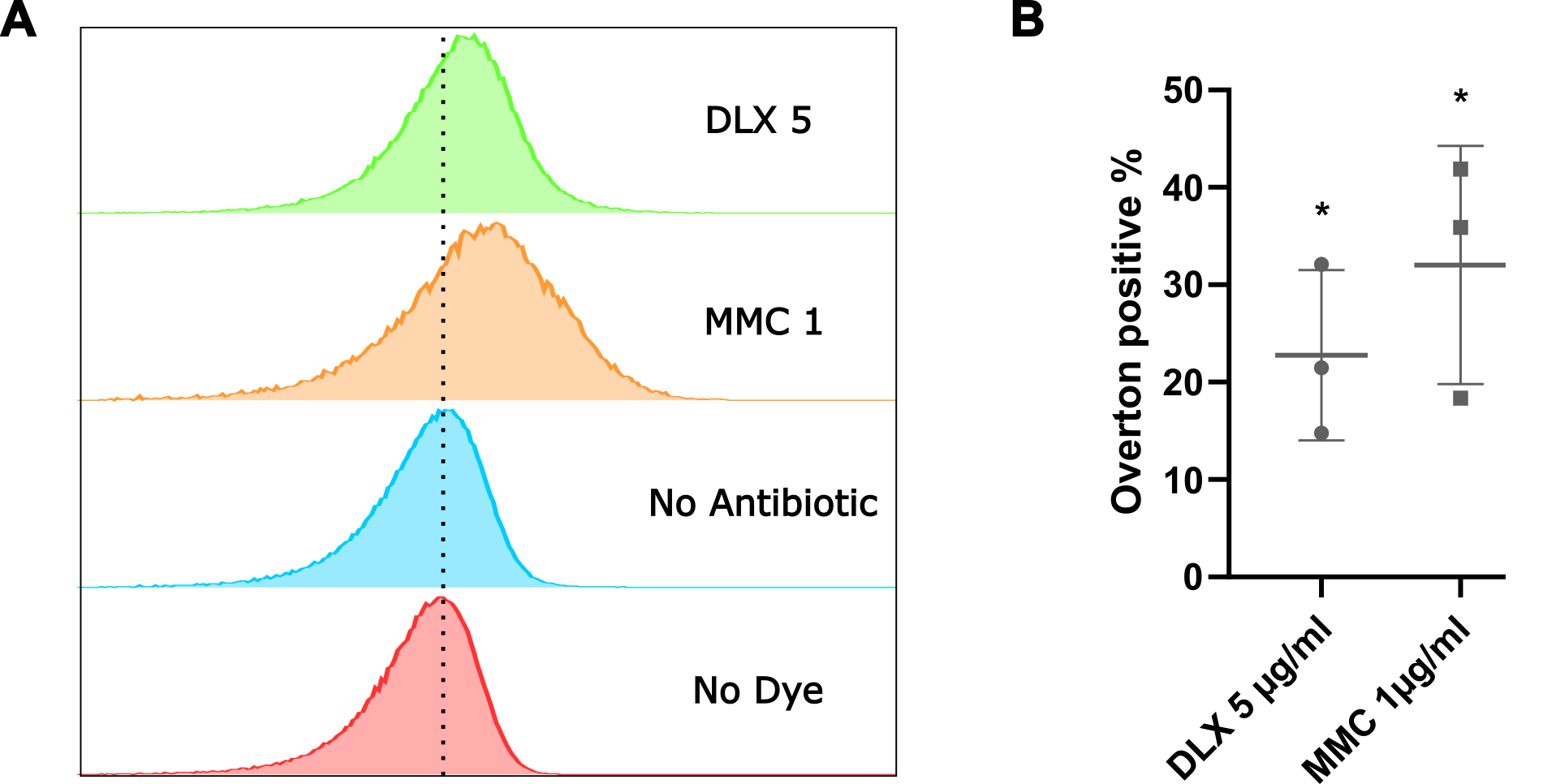

Supplement: S2 Fig — (A) Representative fluorescence from TUNEL staining of WT cells treated with no antibiotic, 1 µg/mL MMC, or 5 µg/mL DLX, or a no-dye control. (B) The Overton positive percentage (representing the percentage of the population that has increased fluorescence compared to the control no antibiotic population) for the DLX and MMC treated samples. Significance is shown for comparison to a value of 0, as tested by one-sample t-tests (* p < 0.05). (TIFF) [file pgen.1012011.s002.tiff]

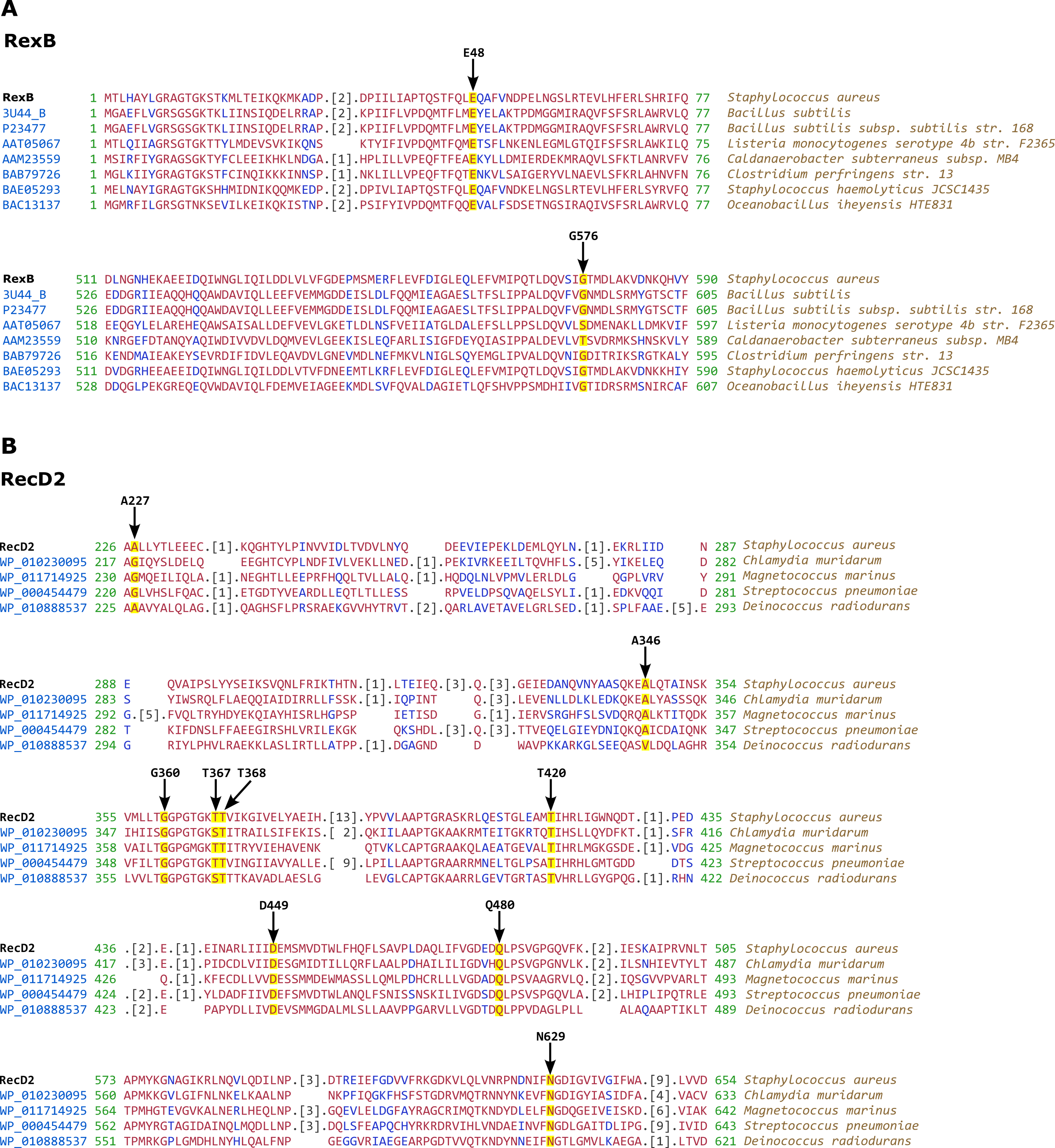

Supplement: S3 Fig — (A, B) Multiple sequence alignments of (A) RexB and (B) RecD2 from the Conserved Domain Database [71,72], where residues in red are highly conserved, and those in blue are not. Arrows denote the position of mutations, which are also highlighted, seen in the initial recA::Tn evolved populations. (TIFF) [file pgen.1012011.s003.tiff]

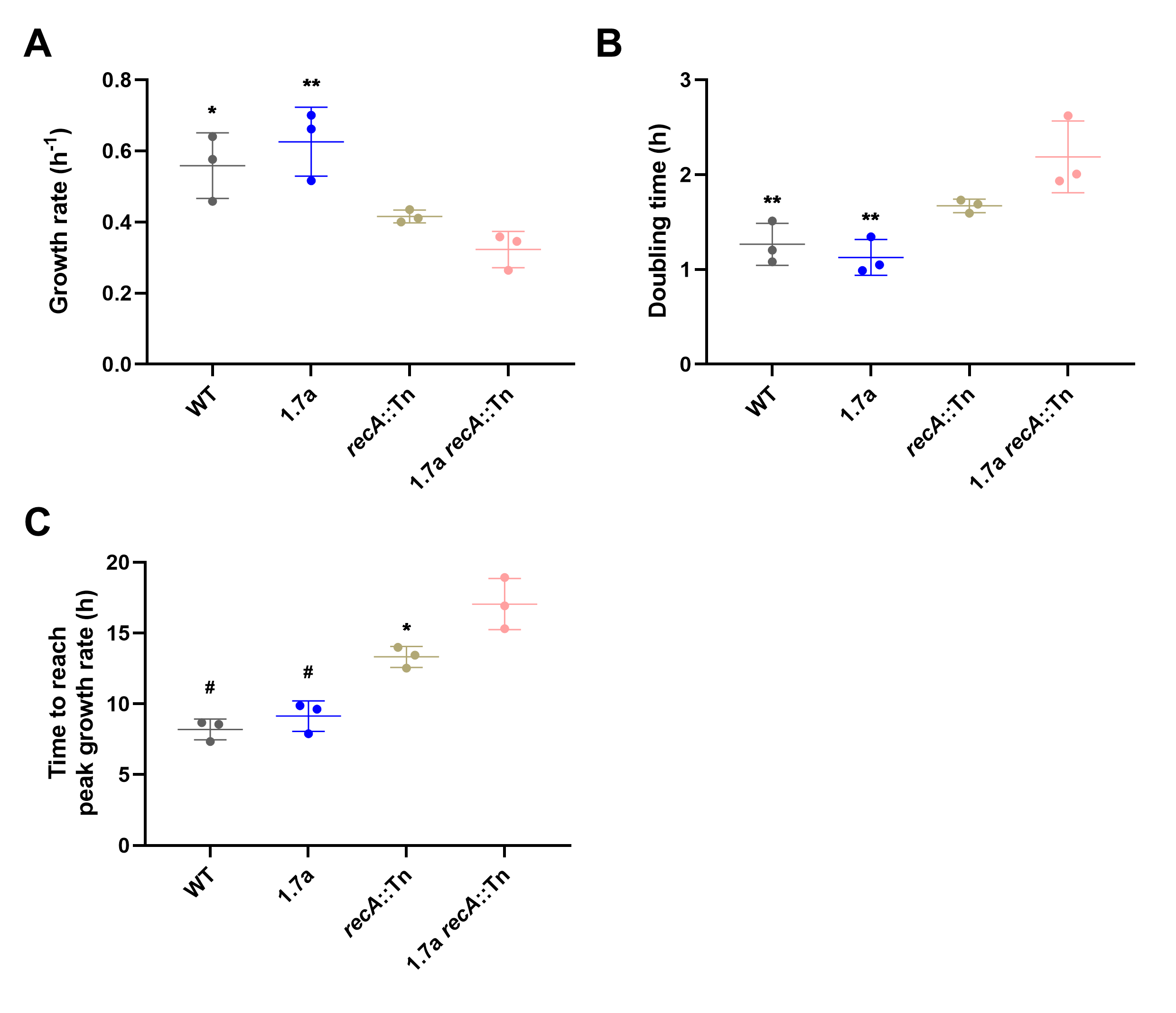

Supplement: S4 Fig — (A) Growth rates, (B) doubling times, and (C) time to reach the peak growth rate are shown for the WT, and 1.7a, and recA::Tn mutants in both backgrounds. These parameters were obtained from growth curves shown in Fig. 3D using Growthcurver [44] for fitting and analysis. Data shown are the means ± standard deviation for three independent biological replicates. Significance is shown for comparison to the 1.7a recA::Tn strain, as tested by a one-way ANOVA with Dunnett’s test for multiple comparisons (* p < 0.05, ** p < 0.01, # p < 0.0001). (TIF) [file pgen.1012011.s004.tif]

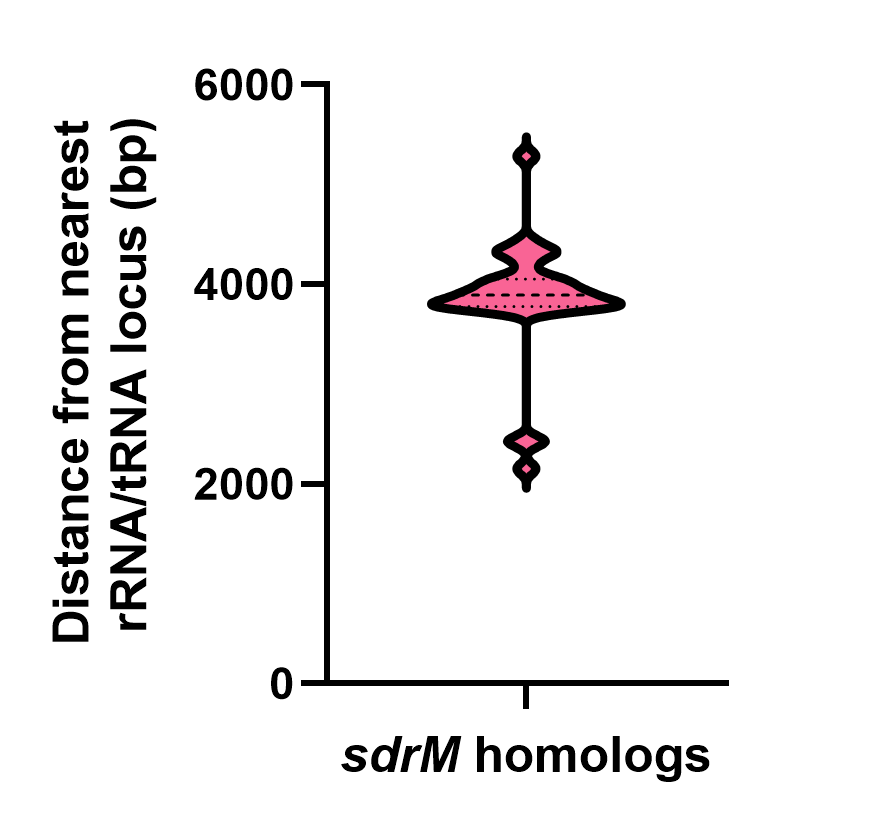

Supplement: S5 Fig — Homologs of sdrM were identified using BLASTP [68] against the ClusteredNR database on NCBI using default settings. Shown are the distances of the sdrM homologs to the nearest rRNA-tRNA cluster. (TIF) [file pgen.1012011.s005.tif]

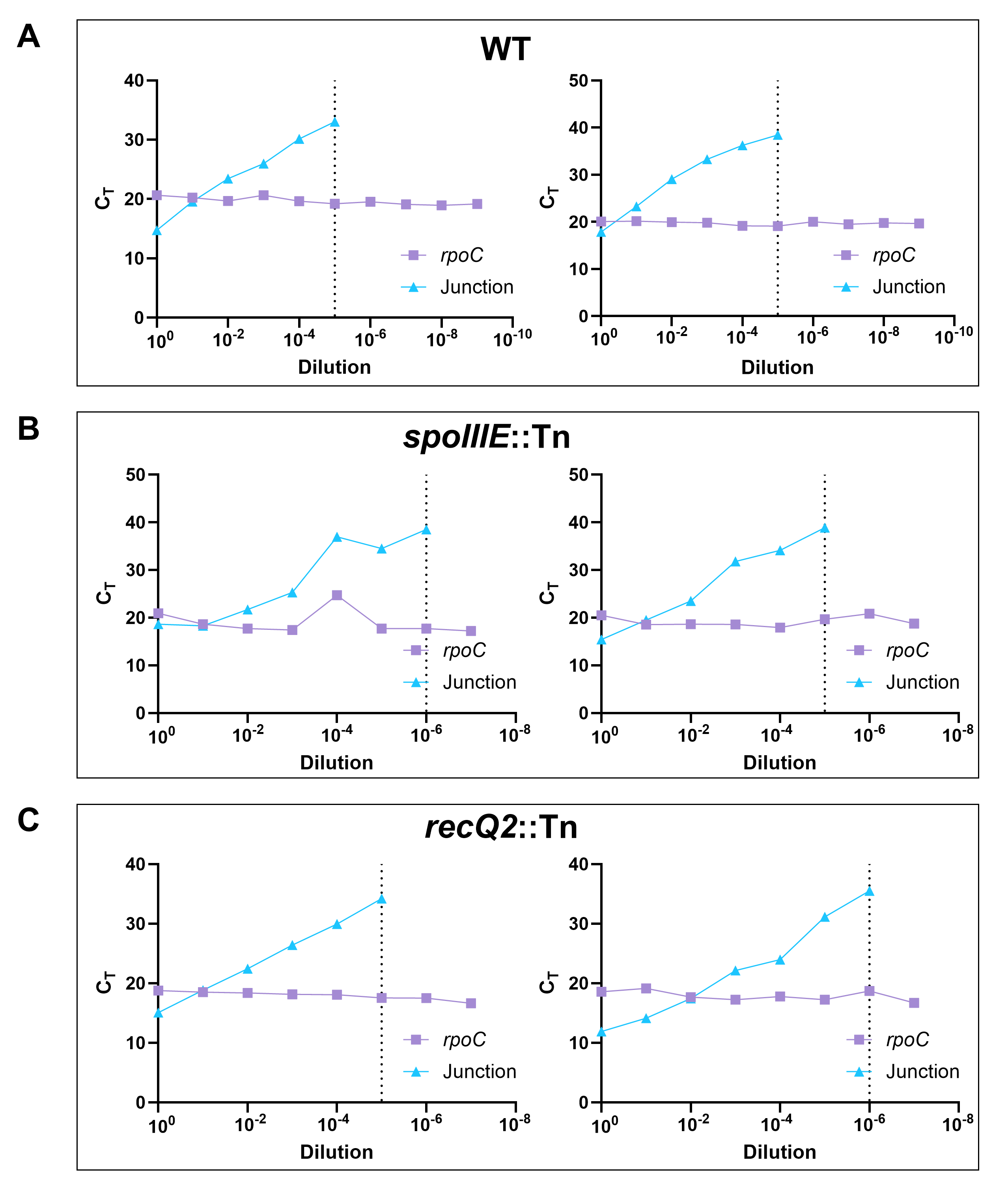

Supplement: S6 Fig — qPCR CT values for the control housekeeping gene rpoC and the respective novel junction present in two different evolved DLX resistant populations from (A) WT, (B) spoIIIE::Tn, and (C) recQ2::Tn, measured in a 10-fold dilution series where genomic DNA from the evolved population was diluted in genomic DNA from the parental strain. (TIF) [file pgen.1012011.s006.tif]

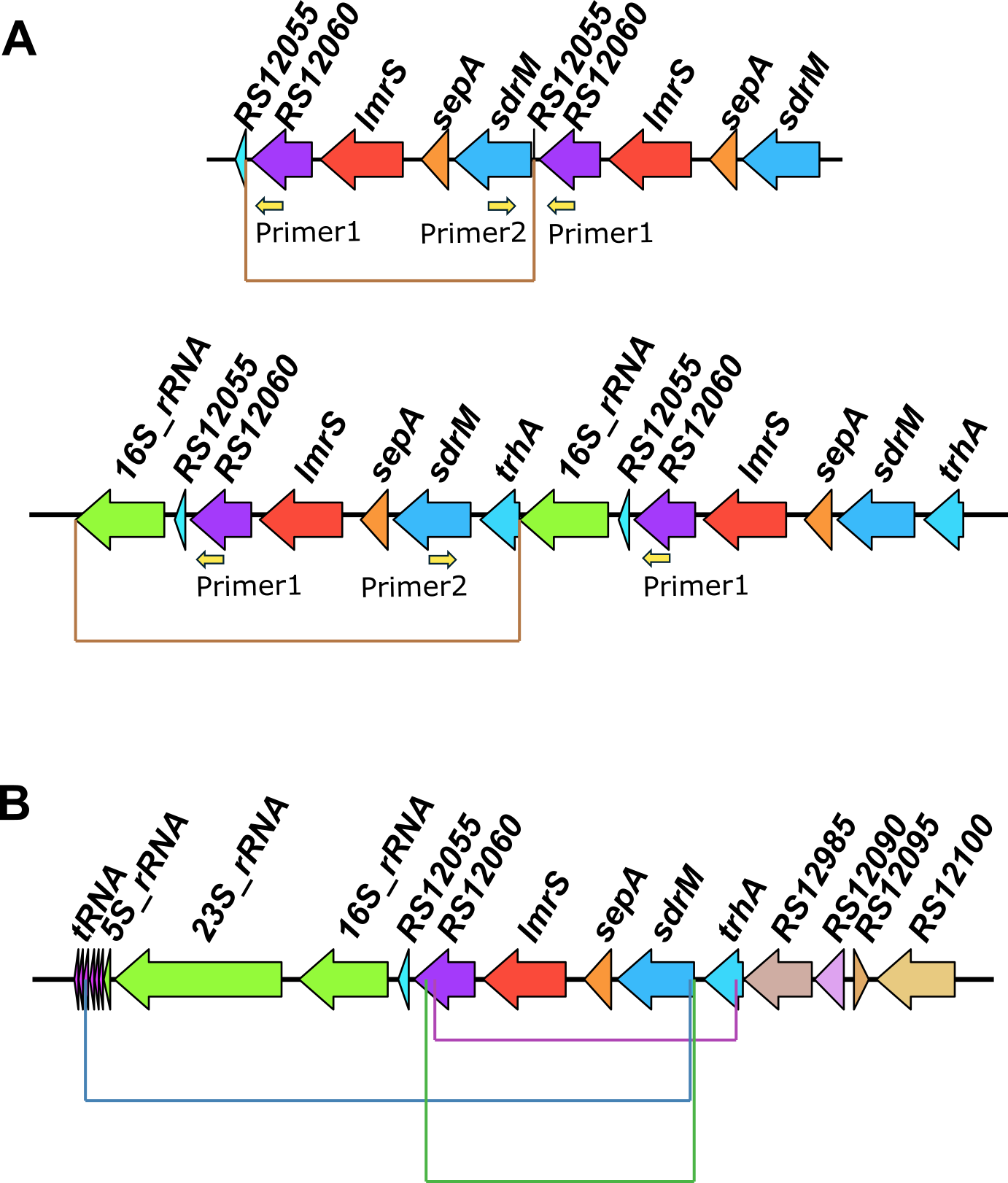

Supplement: S7 Fig — (A) Inverse-PCR like strategy to detect novel junctions present around sdrM. Shown are two hypothetical amplified fragments (outlined by the brown lines). We designed outward facing primers (Primer 1 and Primer 2) binding to sdrM and B7H15_RS12060. If a duplicated or amplified fragment is present in that region, the novel junction should get amplified by the PCR primers. (B) Three putative duplicated or amplified fragments identified in the WT strain using the inverse PCR like strategy. (TIFF) [file pgen.1012011.s007.tiff]

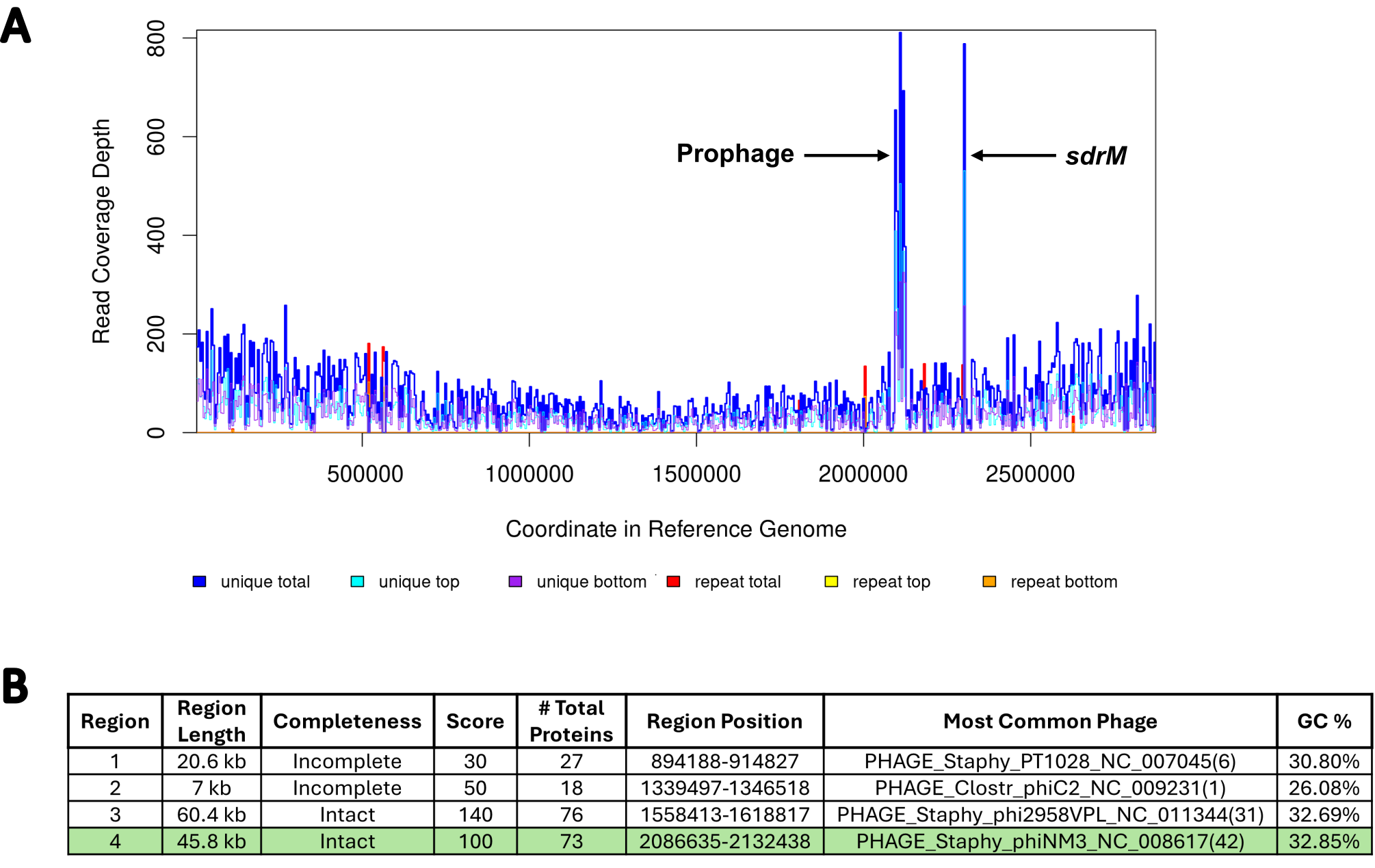

Supplement: S8 Fig — (A) Genomic coverage from whole-genome sequencing of a WT evolved population showing increased coverage for the 43 kb prophage region as well as the sdrM locus. (B) The JE2 genome was analyzed using PHASTER [35] to identify prophage regions, and the PHASTER output is shown. Region 4 (in green) encodes an intact prophage whose coordinates match the region that shows increased sequencing coverage depth in the evolved DLX resistant populations. (TIFF) [file pgen.1012011.s008.tiff]

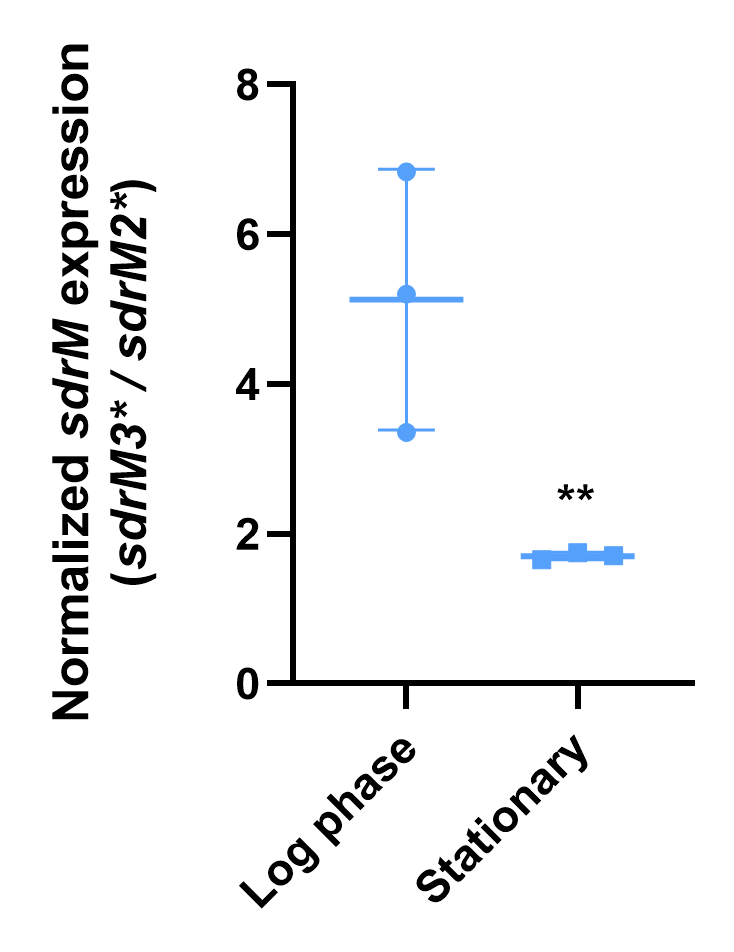

Supplement: S9 Fig — Expression of sdrM was measured in log phase (OD600 = 0.5) and stationary phase (overnight cultures) of the previously reported sdrM2* and sdrM3* allele-replacement mutants [16], using qPCR where the rpoC gene was used as the control housekeeping gene. The sdrM2* mutant has the A268S coding sequence mutation, while the sdrM3* mutant has the A268S mutation as well as a C to G change at position -164 (upstream of sdrM). Data shown are the means ± standard deviation for three independent biological replicates. Significance is shown for comparison to 1, as tested by a one-sample t-test (** p < 0.01). (TIF) [file pgen.1012011.s009.tif]

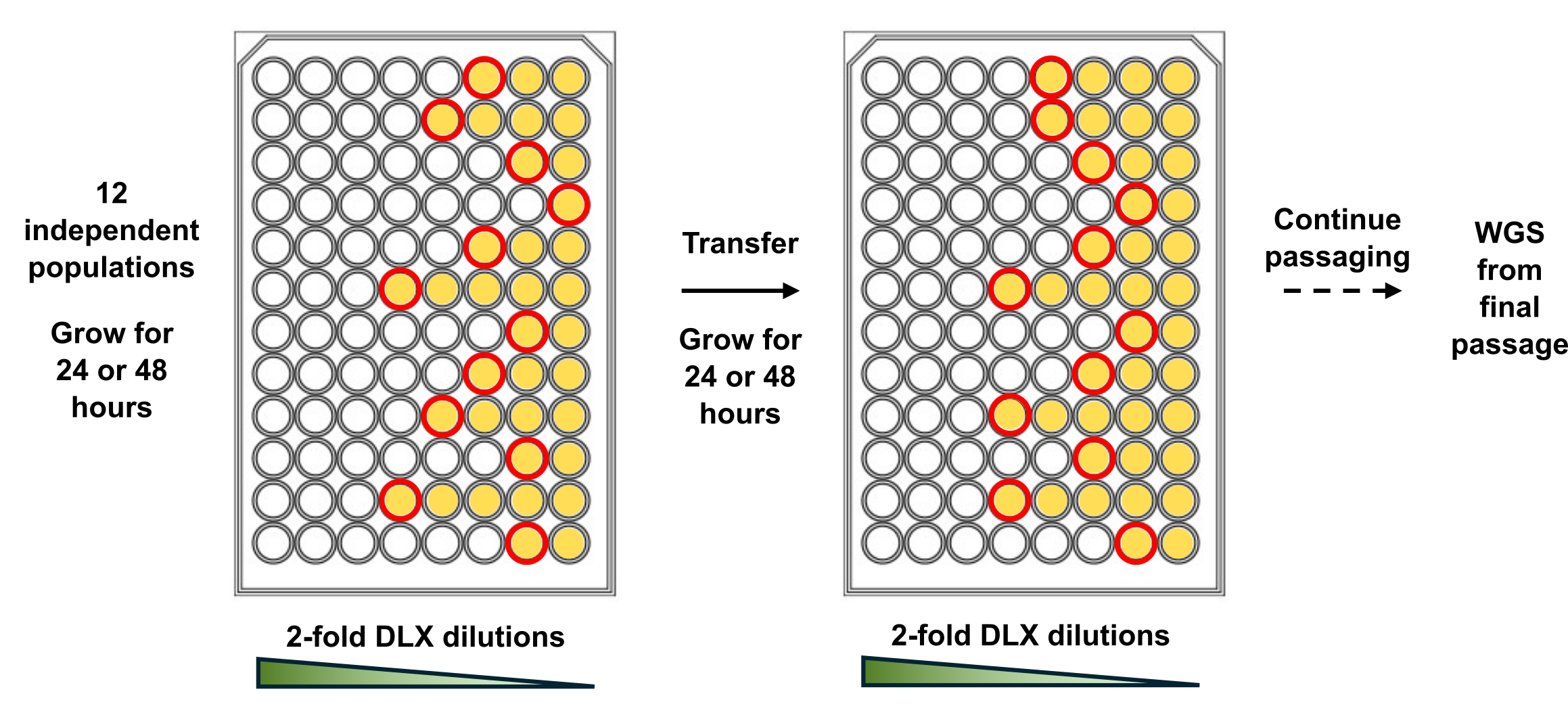

Supplement: S10 Fig — 12 independent populations of the strain to be evolved were inoculated in a 2-fold dilution series of DLX and grown for 24 or 48 hours depending on the specific strains being tested. Populations from the well with the highest DLX concentration that showed growth were transferred to the next passage. Wells showing growth are denoted in yellow, and of those, the ones in the highest DLX concentration are marked with the red circles. Populations from these red-circled wells were propagated to the next passage. After the specified number of passages, the terminal passages from the wells with the highest DLX concentration that showed growth were sent for WGS. (TIFF) [file pgen.1012011.s010.tiff]

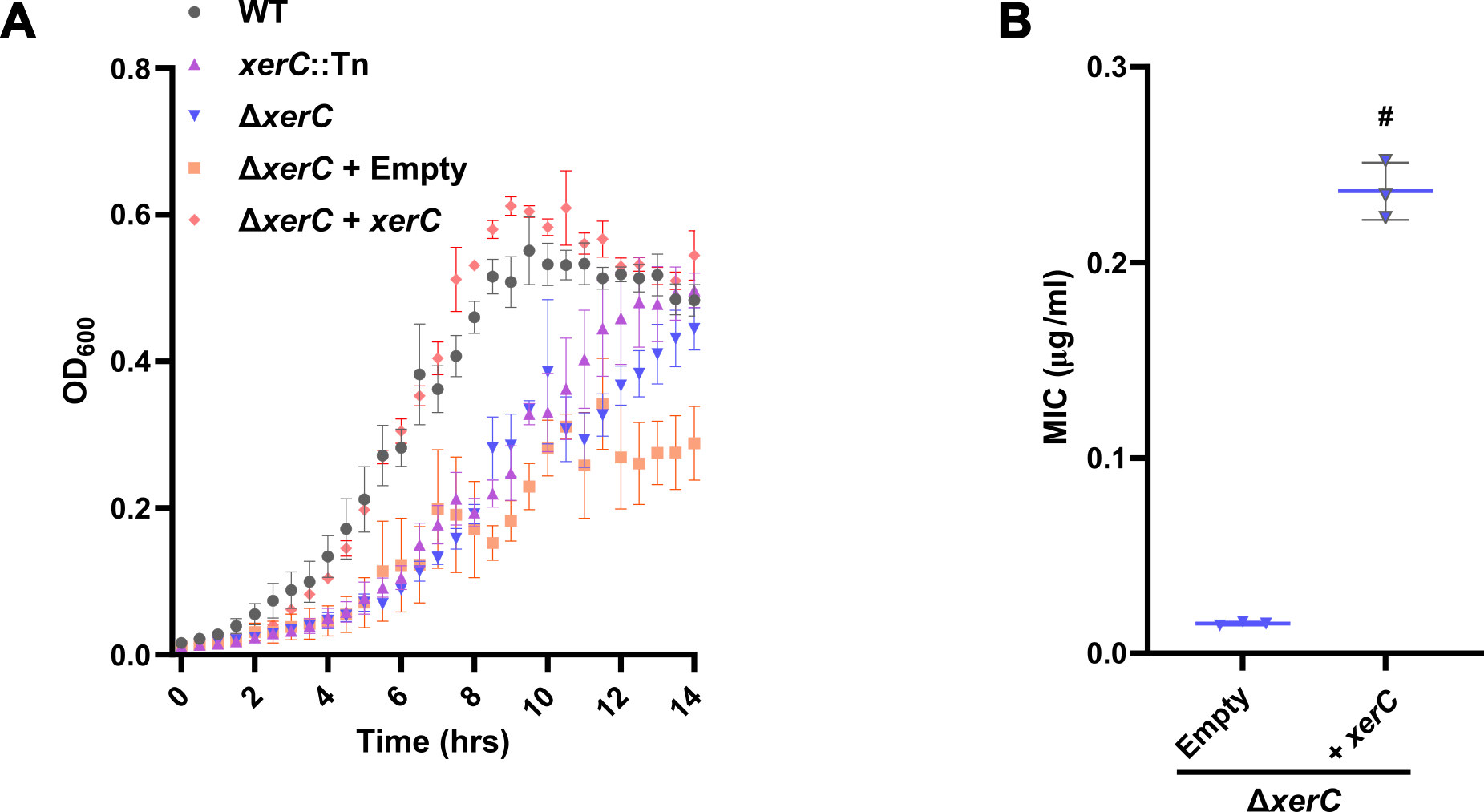

Supplement: S11 Fig — (A) Overnight cultures of the indicated strains were diluted 1:100 in modified M63 medium and OD600 was measured every 30 minutes. Data shown are mean ± SEM of three independent biological replicates. (B) DLX MICs of the ΔxerC mutant containing either the empty pKK30 plasmid or one expressing xerC from its native promoter. Data shown are mean ± standard deviation for three independent biological replicates. Significance is shown for comparison to the empty vector control, as tested by an unpaired t-test (# p < 0.0001). (TIFF) [file pgen.1012011.s011.tiff]

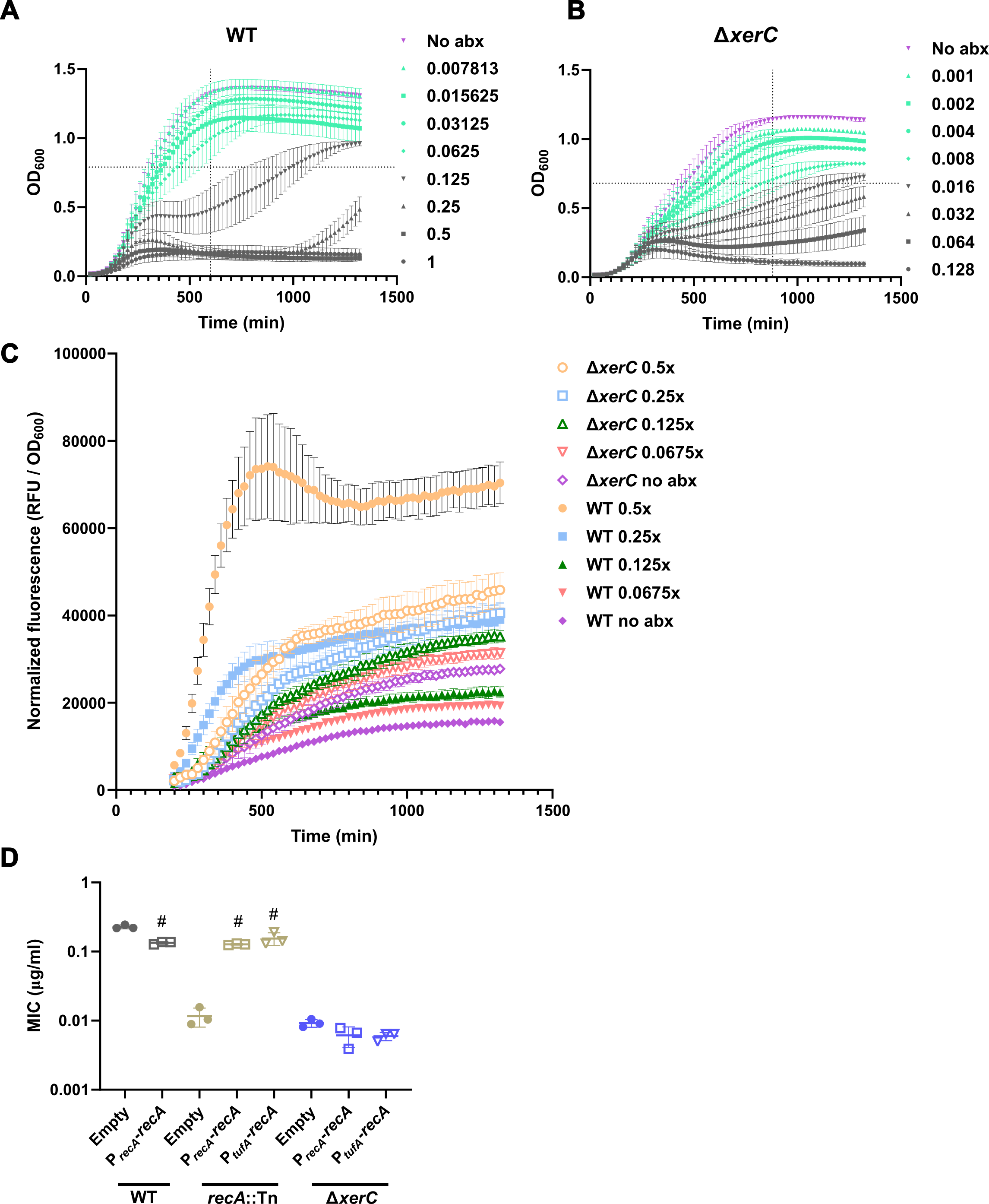

Supplement: S12 Fig — (A-C) WT and the ΔxerC mutant were grown in a dilution series of DLX, and fluorescence (excitation 560 nm; emission 587 nm) and A600 measured every 20 minutes. Shown are the mean ± SEM of three independent biological replicates. A600 of (A) WT and (B) the ΔxerC mutant is shown. The horizontal dotted line represents 60% of the mean carrying capacity for the no DLX condition, and the vertical dotted line represents the time at which the growth without DLX first reaches the mean carrying capacity. The growth curves in green represent the DLX concentrations below the respective IC-40, and the curves in grey represent the DLX concentrations equal to or greater than the respective IC-40. (C) Normalized fluorescence (relative to the OD600) for WT and the ΔxerC mutant grown in 0.5x, 0.25x, 0.125x, 0.0675x the respective IC-40, or without DLX. (A-C) Data shown are the mean ± SEM of four independent biological replicates. (D) DLX MICs of the WT, recA::Tn, and ΔxerC containing either the pKK30 empty vector, or one containing PrecA::recA or PtufA::recA (for recA::Tn, and ΔxerC). Data shown are the mean ± standard deviation of three independent biological replicates. Significance is shown for comparison to the respective empty vector control, as tested by a one-way ANOVA followed by a Tukey’s test for multiple comparisons (# p < 0.0001). (TIFF) [file pgen.1012011.s012.tiff]
