# Supplementary material for: Modulators of gene amplification alter evolution of antibiotic resistance in Staphylococcus aureus
Source: PLoS Genet. 2025 Dec 31;21(12):e1012011. doi: 10.1371/journal.pgen.1012011 (PMC12795462; doi:10.1371/journal.pgen.1012011)
Supplement: S9 Table — (PDF) [file pgen.1012011.s021.pdf]

Name	Description	Source
**Strains**		
S. aureus		
JE2	*Staphylococcus aureus* subsp. *aureus* USA300_FPR3757 (CA-MRSA)-JE2	[1]
RN4220	Restriction modification deficient *S. aureus*; used to shuttle pKK30 derivatives into JE2	[2]
SB514	1.7a (evolved DLX resistant mutant strain)	[3]
SB616	JE2 $lexA^{S130A}$	This study
SB617	JE2 $lexA^{G94E}$	This study
NE805	JE2 *recA*::Tn	[1]
NE331	JE2 *rexA*::Tn	[1]
NE1427	JE2 *recD2*::Tn	[1]
NE959	JE2 *sbcD*::Tn	[1]
NE324	JE2 *recX*::Tn	[1]
NE1673	JE2 *B7H15_RS12095*::Tn	[1]
NE883	JE2 *xerC*::Tn	[1]
NE445	JE2 *umuC*::Tn	[1]
NE1344	JE2 *recG*::Tn	[1]
NE346	JE2 *dinG*::Tn	[1]
NE972	JE2 *recQ*::Tn	[1]
NE1146	JE2 *B7H15_RS12090*::Tn	[1]
NE145	JE2 *uvrA*::Tn	[1]
NE1794	JE2 *ruvX*::Tn	[1]
NE555	JE2 *recF*::Tn	[1]
NE711	JE2 *B7H15_RS09290*::Tn	[1]
NE341	JE2 *rarA*::Tn	[1]
NE458	JE2 *xseA*::Tn	[1]
NE1679	JE2 *ssbB*::Tn	[1]
NE1830	JE2 *recT*::Tn	[1]
NE1528	JE2 *recQ2*::Tn	[1]
NE1866	JE2 *dinB*::Tn	[1]
NE188	JE2 *mfd*::Tn	[1]
NE97	JE2 *B7H15_RS12085*::Tn	[1]
NE1760	JE2 *B7H15_RS08985*::Tn	[1]
NE993	JE2 *trhA*::Tn	[1]
NE1689	JE2 *B7H15_RS10630*::Tn	[1]
NE1616	JE2 *sepA*::Tn	[1]
NE100	JE2 *B7H15_RS03470*::Tn	[1]
NE348	JE2 *spoIIIE*::Tn	[1]
NE1529	JE2 *B7H15_RS12060*::Tn	[1]
NE1915	JE2 *B7H15_RS03710*::Tn	[1]
NE22	JE2 *polA*::Tn	[1]
NE152	JE2 *B7H15_RS12505*::Tn	[1]

NE531	JE2 *sdrM*::Tn	[1]
NE1012	JE2 *rexB*::Tn	[1]
NE486	JE2 *noc*::Tn	[1]
NE1085	JE2 *scpB*::Tn	[1]
NE243	JE2 *B7H15_RS08105*::Tn	[1]
NE761	JE2 *nth*::Tn	[1]
NE1028	JE2 *B7H15_RS08555*::Tn	[1]
SB618	Evolved *recA*::Tn isolate RecA1.P14a (R1)	This study
SB619	Evolved *recA*::Tn isolate RecA2.P13a (R2)	This study
SB620	Evolved *recA*::Tn isolate RecA3.P14a (R3)	This study
SB622	1.7a *recA*::Tn	This study
SB482	JE2 $recD2^{A227E}$	This study
SB483	JE2 $rexB^{G576D}$	This study
SB484	JE2 $P_{090/095}^{G\rightarrow A}$ (at position 2,306,688)	This study
SB486	JE2 $recD2^{A227E}$ *recA*::Tn	This study
SB487	JE2 $rexB^{G576D}$ *recA*::Tn	This study
SB489	JE2 $P_{090/095}^{G\rightarrow A}$ *recA*::Tn	This study
SB621	Evolved DLX-resistant *xerC*::Tn mutant XC5_7	This study
SB644	Δ*xerC*	This study
SB675	$P_{090/095}^{G\rightarrow A}$ *sdrM*::Tn	This study
E. coli		
DH5α-λpir	DH5α lysogenized with λpir; host for pKK30	[4]
IM08B	For plasmid transfer into *S. aureus*	[5]
**Plasmids**		
pIMAY*	*S. aureus* allelic exchange plasmid; Cm^R	[6]
pKK30	Expression vector for *S. aureus*; Tmp^R	[7]
pKAN	*bursa aurealis* replacement vector; Kan^R	[8]
pKM16	Fluorescent reporter (P_{sarA1}::*dsRed3.T3*); Cm^R	[9]
pSB627	pIMAY*-$recD2^{A227E}$	This study
pSB626	pIMAY*-$rexB^{G576D}$	This study
pSB628	pIMAY*- $P_{090/095}^{G\rightarrow A}$	This study
pSB623	pIMAY*-*lexA*	This study
pSB624	pIMAY*-$lexA^{S130A}$	This study
pSB625	pIMAY*-$lexA^{G94E}$	This study
pSB643	pIMAY*-Δ*xerC*	This study
pSB469	pKK30-*recA*	This study
pSB598	pKK30-*xerC*	This study
pSB600	pKK30-*RS12060*	This study
pSB654	pKK30- P_{tufA}::*recA*	This study
pSB639	pKK15A; Kan^R	This study
pSB640	pKK15A P_{sarA1}::*dsRed3.T3*	This study
pSB672	pKK15A P_{recA}::*dsRed3.T3*	This study

Cm^R: chloramphenicol resistant; Tmp^R: trimethoprim resistant; Kan^R: kanamycin resistant

REFERENCES

1. Fey PD, Endres JL, Yajjala VK, Widhelm TJ, Boissy RJ, Bose JL, Bayles KW. A genetic resource for rapid and comprehensive phenotype screening of nonessential *Staphylococcus aureus* genes. mBio. 2013;4(1):e00537-12. PubMed PMID: 23404398.

2. De Azavedo J, Foster TJ, Hartigan PJ, Arbuthnott JP, O'Reilly M, Kreiswirth BN, Novick RP. Expression of the cloned toxic shock syndrome toxin 1 gene (tst) in vivo with a rabbit uterine model. Infection and Immunity. 1985;50(1):304-9. PubMed PMID: 4044040.

3. Silva KPT, Sundar G, Khare A. Efflux pump gene amplifications bypass necessity of multiple target mutations for resistance against dual-targeting antibiotic. Nature Communications. 2023;14(1):3402. PubMed PMID: 37296157.

4. Dunn AK, Martin MO, Stabb EV. Characterization of pES213, a small mobilizable plasmid from *Vibrio fischeri*. Plasmid. 2005;54(2):114-34. PubMed PMID: 16122560.

5. Monk IR, Tree JJ, Howden BP, Stinear TP, Foster TJ. Complete bypass of restriction systems for major *Staphylococcus aureus* lineages. mBio. 2015;6(3):e00308-15. PubMed PMID: 26015493.

6. Schuster CF, Howard SA, Gründling A. Use of the counter selectable marker PheS* for genome engineering in *Staphylococcus aureus*. Microbiology. 2019;165(5):572-84. PubMed PMID: 30942689.

7. Krute CN, Krausz KL, Markiewicz MA, Joyner JA, Pokhrel S, Hall PR, Bose JL. Generation of a stable plasmid for in vitro and in vivo studies of *Staphylococcus* species. Applied and Environmental Microbiology. 2016;82(23):6859-69. PubMed PMID: 27637878.

8. Bose JL, Fey PD, Bayles KW. Genetic tools to enhance the study of gene function and regulation in *Staphylococcus aureus*. Applied and Environmental Microbiology. 2013;79(7):2218-24. PubMed PMID: 23354696.

9. Mlynek KD, Bulock LL, Stone CJ, Curran LJ, Sadykov MR, Bayles KW, Brinsmade SR. Genetic and biochemical analysis of CodY-mediated cell aggregation in *Staphylococcus aureus* reveals an interaction between extracellular DNA and polysaccharide in the extracellular matrix. Journal of Bacteriology. 2020;202(8). PubMed PMID: 32015143.
