# Supplementary material for: Modulators of gene amplification alter evolution of antibiotic resistance in Staphylococcus aureus
Source: PLoS Genet. 2025 Dec 31;21(12):e1012011. doi: 10.1371/journal.pgen.1012011 (PMC12795462; doi:10.1371/journal.pgen.1012011)
Supplement: S10 Table — (PDF) [file pgen.1012011.s022.pdf]

Primer Description	Primer Sequence*
Amplify *rexB*^{G576D} for pIMAY* F	TATCGATAAGCTTGATATCGAATTTAAACATAATTTCAATCCTGAAAATAC
Amplify *rexB*^{G576D} for pIMAY* R	TGGAGCTCCACCGCGGTGGCCTCAACTAGTACAGCTGTTTTAC
Check pIMAY*-*rexB*^{G576D} integration F	ATACAACATCGTTTGTCGGTTT
Check pIMAY*-*rexB*^{G576D} integration R	TTGCGCGTCAGTCCAAAT
Sanger sequencing for *rexB*^{G576D}	GGCGTAAAGCGCATAAACTG
Amplify *recD2*^{A227E} for pIMAY* F	TATCGATAAGCTTGATATCGAAAAATTGATGACACAGATAGTC
Amplify *recD2*^{A227E} for pIMAY* R	TGGAGCTCCACCGCGGTGGCAACTCAACTTAAACATGTATTAAC
Check pIMAY*-*recD2*^{A227E} integration F	CGGTACCACCCTAGTTATAAATGC
Check pIMAY*- *recD2*^{A227E} integration R	ATGCAAGATACAATTTGGGCTTAG
Sanger sequencing for *recD2*^{A227E}	CTTGGAATTGCAACTTGTTCATTG
Amplify $P_{090/095}^{G\rightarrow A}$ for pIMAY* F	TATCGATAAGCTTGATATCGCTATAAAAACTTAGTATTCCAGTTG
Amplify $P_{090/095}^{G\rightarrow A}$ for pIMAY* R	TGGAGCTCCACCGCGGTGGCTTTAATTTCACTTGTAGGAATTTTG
Check pIMAY*- $P_{090/095}^{G\rightarrow A}$ integration F	TCTAATGACGCTACCTTCTCTTC
Check pIMAY*- $P_{090/095}^{G\rightarrow A}$ integration R	TGTATTCGTGGTCTTCAGTATCG
Sanger sequencing for $P_{090/095}^{G\rightarrow A}$	TTCCGACCATTAACGCTGTAG
Mutagenesis of *lexA* to mutant allele S130A F	TAATATACCAGCCTCAATCATAGCGTCGCCTACGACGTTTAATATG
Mutagenesis of *lexA* to mutant allele S130A R	CATATTAAACGTCGTAGGCGACGCTATGATTGAGGCTGGTATATTA
Mutagenesis of *lexA* to mutant allele G94E F	TTTCTACTGCGGTAATAGGAACCTCTGCTGTGACTTTACCAATAAC
Mutagenesis of *lexA* to mutant allele G94E R	GTTATTGGTAAAGTCACAGCAGAGGTTCCTATTACCGCAGTAGAAA
Amplify *lexA* for pIMAY* F	TGGAGCTCCACCGCGGTGGCTTACATTTCGCGGTACAAAC
Amplify *lexA* for pIMAY* R	TATCGATAAGCTTGATATCGATGAGAGAATTAACAAAACGAC
Check pIMAY*-lexA integration_F	ATAATGGCTCGCTCCTGTAAAT
Check pIMAY*-lexA integration_R	TCCCATCTCTTTGTTCGCTATT
Sanger sequencing for both *lexA* mutant alleles	TTTCGCGGTACAAACCAATTAC
Amplify upstream region of *xerC* for pIMAY* F	CGAATTGGAGCTCCACCGCGGTGGCATGTAGAAAGCGCAGCTAG
Amplify upstream region of *xerC* for pIMAY* R	TACTCATGTTTCATGATTCAATACATTTACCCTC
Amplify downstream region of *xerC* for pIMAY* F	TGTATTGAATCATGAAACATGAGTAATACAACATTAC
Amplify downstream region of *xerC* for pIMAY* R	GACGGTATCGATAAGCTTGATATCGTTTAGCATCATTTTGTCCAAC

Integration and seq primer for *ΔxerC* F	AAGACTGGGAAACGTCCTTATG
Integration and seq primer for *ΔxerC* R	TCCTCAGTAGGTGGTTCTTCT
Gibson assembly for pKK30-*recA* F	TTGGATAACGATCGTCAAAA
Amplify *tufA* promoter to replace *recA* promoter in pKK30 P_{recA}::*recA* F	CAAGCAAAGTGACAGGCGATACATCATTACGTTCAAACAC
Amplify *tufA* promoter to replace *recA* promoter in pKK30 P_{recA}::*recA* R	TTTTGACGATCGTTATCCAACAAGAGTTCCTCCTTCAAAAG
Clone *recA* promoter to replace *sarA1* promoter in pKK15a P_{recA}:: *dsRed3.T3* F	TTCGAAAGGACAGATTGTGTTTGTATTATCGATAAAAATATAAGCAC
Clone *recA* promoter to replace *sarA1* promoter in pKK15a P_{recA}:: *dsRed3.T3* R	ACATCTTCTGTATTATCCATAGCGAGACCTCCTAATTG
Check transposon insertion for *recF*::Tn _F	TGAAACACGTCGCGGTAAA
Check transposon insertion for *recF*::Tn _R	CATCTCGATGTGGTCCGAATAA
Check transposon insertion for *xerC*::Tn _F	TTCTTAAGTCATCAACGAGTAA
Check transposon insertion for *xerC*::Tn _R	AAGCGTTACTTGCCCATCTC
Check transposon insertion for *recG*::Tn _F	CGCGTTTATGGGCTTGATTG
Check transposon insertion for *recG*::Tn _R	GTATCGGCGTTGCTGTCATA
Check transposon insertion for *sbcD*::Tn _F	GGAGAATTAGGCGGCATGTT
Check transposon insertion for *sbcD*::Tn _R	ACCTCTCTTTACCATCGTGATTT
Check transposon insertion for *recJ*::Tn _F	CCATCCTACTTGTGCACCTAA
Check transposon insertion for *recJ*::Tn _R	CTTCAATCTTCATTGCCGTT
Check transposon insertion for *recQ*::Tn _F	TCGTAATGTGCTTGGTGTCTTA
Check transposon insertion for *recQ*::Tn _R	TGGTAACTCGGCCTGAAATC
Check transposon insertion for *recX*::Tn _F	GTTGCGTGTACTTCTTTCGATTT
Check transposon insertion for *recX*::Tn _R	TGATCCAGTGCCGAAGATTAC
Check transposon insertion for *uvrA*::Tn _F	TGGGTCAGGTAAATCGTCATTAG
Check transposon insertion for *uvrA*::Tn _R	AAATCAGAACTCGTCGGTATCC
Check transposon insertion for *dinG*::Tn _F	GGCGTCTCTGTAATCCAAATCA
Check transposon insertion for *dinG*::Tn _R	GATGCCGCTACTACTGCTAAA
Check transposon insertion for *RS09290*::Tn _F	TGTAAGTGAGGAGGGATGTACT
Check transposon insertion for *RS09290*::Tn _R	CAATCGCTTGCTTCGTTCTTC
Check transposon insertion for *rarA*::Tn _F	ACCAAGAGGCATTATCAGAAGAA
Check transposon insertion for *rarA*::Tn _R	AGTTGTAGCACCGATCAAGAC
Check transposon insertion for *xseA*::Tn _F	GCACCTGTACTCGCTGTTAAA
Check transposon insertion for *xseA*::Tn _R	CTGGCGTTAAAGGTCACGAA
Check transposon insertion for *ssbB*::Tn _F	CAAAGTGTGTTTGTCCGTCTTTAT
Check transposon insertion for *ssbB*::Tn _R	AATCGTAATTGTCGGGAGACTG
Check transposon insertion for *recT*::Tn _F	CTTGCTTGAATGTATCGCCTTTAT
Check transposon insertion for *recT*::Tn _R	TTCACCAAGTAATGCCATGAAAC
Check transposon insertion for *recQ2*::Tn _F	AAGCACTCGTTGCGACTATAA
Check transposon insertion for *recQ2*::Tn _R	AGCGGTTGTCTTAGCATTGA
Check transposon insertion for *RS12085*::Tn _F	CCATTACCATTCGGCGTTTC
Check transposon insertion for *RS12085*::Tn _R	TGAAGAGAAGGTAGCGTCATTAG
Check transposon insertion for *dinB*::Tn _F	CTTCATCATCATTCACGTCTGTTG
Check transposon insertion for *dinB*::Tn _R	AGCATCTGCAGGTGTTTCTTA

Check transposon insertion for *mfd*::Tn _F	CTGCGTTAGCTCAAGGTAAGA
Check transposon insertion for *mfd*::Tn _R	CGCTGCGTTTCAACATCAA
Check transposon insertion for *ruvX*::Tn _F	AGCACCTTCTTCAGCTAAGATAAC
Check transposon insertion for *ruvX*::Tn _R	GGCACAAGGATTAGACACACTC
Check transposon insertion for *RS08985*::Tn _F	AGCGCCAATAACATCTTCTACT
Check transposon insertion for *RS08985*::Tn _R	AGTGATCCGACATTGTCCATTTA
Check transposon insertion for *RS06660*::Tn _F	TTTGGTGGCGGTGTATTAGG
Check transposon insertion for *RS06660*::Tn _R	TGCAGAACGACCAGGTATTG
Check transposon insertion for *trhA*::Tn _F	CAACCTATCCAACCACCTACAA
Check transposon insertion for *trhA*::Tn _R	AATGCGGCATCTCATGGT
Check transposon insertion for *RS12060*::Tn _F	TCCAGGTGGAAGATCGAGTAT
Check transposon insertion for *RS12060*::Tn _R	GGTAAAGGTGGTGTCGGTAAA
Check transposon insertion for *RS03470*::Tn _F	CCAGCTTGACGACCTTTCATA
Check transposon insertion for *RS03470*::Tn _R	GGCGTACGTATACAGCCTTATC
Check transposon insertion for *sepA*::Tn _F	ACGTTGTTGCAACTGTGTAAG
Check transposon insertion for *sepA*::Tn _R	TTTGTACTTTCTGGTGCGATTT
Check transposon insertion for *RS10630*::Tn _F	TCTCTGTCGCTTGTTGTTCTG
Check transposon insertion for *RS10630*::Tn _R	AGGGTATGTCGTGTGCAATG
Check transposon insertion for *RS03710*::Tn _F	TGCAGAAATGAAACTTGCTGTC
Check transposon insertion for *RS03710*::Tn _R	CTACAGCTATAGTGACGATGATT
Check transposon insertion for *RS12505*::Tn _F	CTTCTCGTCCAGCATCTGTT
Check transposon insertion for *RS12505*::Tn _R	GTGATTCAATCGCATCGGTTATT
Check transposon insertion for *polA*::Tn_F	TCTGGCGTCCACCTTTATATTC
Check transposon insertion for *polA*::Tn_R	TGGAGTGCTCATCAAGAACAA
Check transposon insertion for *recA*::Tn_F	TCGGTAAAGGTGCCGTAATG
Check transposon insertion for *recA*::Tn_F	ACGTAACGCTTGTGACATTAAAC
Check transposon insertion for *rexA*::Tn_F	GGTGACACCATACGAAGAAGAA
Check transposon insertion for *rexA*::Tn_F	CTCTTGAACTCGGTTCGTATCT
Check transposon insertion for *RS12090*::Tn _F	TTCCGACCATTAACGCTGTAG
Check transposon insertion for *RS12090*::Tn _R	ACTCTCCATTGAATACGCACTT
Check transposon insertion for *RS12095*::Tn _F	TTCCGACCATTAACGCTGTAG
Check transposon insertion for *RS12095*::Tn _R	TGTATTCGTGGTCTTCAGTATCG
Check transposon insertion for *recD2*::Tn_F	CGGTACCACCCTAGTTATAAATGC
Check transposon insertion for *recD2*::Tn_F	ATGCAAGATACAATTTGGGCTTAG
Check transposon insertion for *umuC*::Tn_F	GTTGTTGCAGATACTAAGCG
Check transposon insertion for *umuC*::Tn_R	AAAGCATTGAACACCATAACCG
Check transposon insertion for *RS08105*::Tn _F	GACCTCTTACCCGTTCATCAC
Check transposon insertion for *RS08105*::Tn _R	TCAATAGATGGTGGAGCGATTAG
Check transposon insertion for *RS08555*::Tn _F	GGTGTCATCGTATTCTCACCTC
Check transposon insertion for *RS08555*::Tn _R	TGCGCAAAGATGACCATTTAAC
Check transposon insertion for *nth*::Tn _F	GTAGGGCTTTGCTTCACTAGAA
Check transposon insertion for *nth*::Tn _R	AGACGAGGCGAATAGTTTGAATA
Gibson assembly for pKK30 F	GCGGCCGCTAGCCTAGGAGC
Gibson assembly for pKK30 and pKK30-*recA* R	ATCGCCTGTCACTTTGCTTGATATATGA

Amplify *xerC* for pKK30 F	CAAGCAAAGTGACAGGCGATAAACTAGAATTAAAGATAAAAAAGAACG
Amplify *xerC* for pKK30 R	GCTCCTAGGCTAGCGGCCGCTCATGTTTCATTCTCCTTTTTC
Amplify *RS12060* for pKK30 F	ATCAAGCAAAGTGACAGGCGATCTATTTATTTGTAGCAGCTATAAC
Amplify *RS12060* for pKK30 R	GAGCTCCTAGGCTAGCGGCCGCTCTCATTTGCTTTTGTAGC
Amplify *recA* for pKK30 F	CAAGCAAAGTGACAGGCGATTTGTATTATCGATAAAAATATAAGCAC
Amplify *recA* for pKK30 R	GCTCCTAGGCTAGCGGCCGCCTATTCTTCGTCAAATAATGAC
Amplify pKK30 to make pKK15A F	CGTAGAAAAGGGAAATAGGC
Amplify pKK30 to make pKK15A R	TTTTTGTGACACTACATACAGC
Amplify *aphA-3* (Kan^R) from pKAN to make pKK15A F	CCAACATAGTGCTAGGGGTTTCAAAATCGG
Amplify *aphA-3* (Kan^R) from pKAN to make pKK15A R	GCCTATTTCCCTTTTCTACGGCTAGGTACTAAAACAATTCATCC
Amplify p15A from pIMAY* to make pKK15A F	TGTATGTAGTGTCACAAAAAAGTAATTTCCTGCATTTGCC
Amplify p15A from pIMAY* to make pKK15A R	AACCCCTAGCACTATGTTGGCACTGATGAG
Amplify pKK15A to insert P_{sarA1}::*dsRed3.T3* F	TGATGGTAAACAATCACCGC
Amplify pKK15A to insert P_{sarA1}::*dsRed3.T3* R	ATCGCCTGTCACTTTGCTTG
Amplify P_{sarA1}::*dsRed3.T3* from pKM16 F	CAAGCAAAGTGACAGGCGATCCATGCCATGTGTAATCCC
Amplify P_{sarA1}::*dsRed3.T3* from pKM16 R	GCGGTGATTGTTTACCATCATGTTCTTTCCTGCGTTATCCC
Check junctions in JE2 WT_F	AATTCGCCCACTATAGGATTGG
Check junctions in JE2 WT_R1	GGAGGCCAATGATTGTGAATTA
Check junctions in JE2 WT_R2	TTTGAAACATTGCCGGAAGAAA
qPCR for junction in DLX resistant population WT2_F	GCAGTTGCAACACTATCTTTACC
qPCR for junction in DLX resistant population WT2_R	CGATGAGTGCTAAGTGTTAGGG
qPCR for junction in DLX resistant population WT3_F	TACTCCAGTACTGTATCTTTACAATCG
qPCR for junction in DLX resistant population WT3_R	AACTGAATGACAATATGTCAACG
qPCR for junction in DLX resistant population recQ2_2_F	TTCTTCACTAAGCGCCACAATA
qPCR for junction in DLX resistant population recQ2_2_R	ACTTGAGTGCAGAAGAGGAAAG
qPCR for junction in DLX resistant population recQ2_3_F	TGTATAAGACACACCACCTAAGAAA
qPCR for junction in DLX resistant population recQ2_3_R	AAAGAAGCGGGTTGTGAAC

qPCR for junction in DLX resistant population spoIIIE1_F	CGATACTGAAGACCACGAATACA
qPCR for junction in DLX resistant population spoIIIE1_R	GTGACACTGCGCCGAAA
qPCR for junction in DLX resistant population spoIIIE2_F	TGTACCAGAAATTGCTACGTAAATC
qPCR for junction in DLX resistant population spoIIIE2_R	TTAAGGCTGAGCTGTGATGG
qPCR for housekeeping gene *rpoC*_F	CTGTGAAAGAATTTTCGGAC
qPCR for housekeeping gene *rpoC*_R	CTTTCACGACGTACTTTAGA
qPCR for *sdrM*_F	GCAATGATCGCAATCGGTAT
qPCR for *sdrM*_R	GGCATAGTTGGCAGTGTTTG
qPCR for *trhA*_F	AGACATACTCACAGACGCAAG
qPCR for *trhA*_R	AATGCGGCATCTCATGGT
qPCR for *RS12085*_F	TTCTTCTAAGTATCCTGCCTTGTC
qPCR for *RS12085*_R	ATTGTGGCGCTTAGTGAAGA
qPCR for *RS12090*_F	AGGTTCGATGTCCAAAGATCAA
qPCR for *RS12090*_R	GCAACTCTAGCTAGCTTATTACCC
qPCR for *RS12095*_F	GCAGTTCCTAAATTAGACAGCAAAG
qPCR for *RS12095*_R	ATGTATTCGTGGTCTTCAGTATCG

*Underlined sequences represent homology for Gibson assembly.
